# SMURF2 inhibits autophagic control of *Mycobacterium tuberculosis* in macrophages

**DOI:** 10.1172/jci.insight.199853

**Published:** 2026-09-08

**Authors:** Priscila C. Campos, Kathryn C. Rahlwes, Victoria A. Ektnitphong, Beatriz R.S. Dias, Kubra F. Naqvi, Samuel Alvarez-Arguedas, Michael U. Shiloh

**Affiliations:** 1 Department of Internal Medicine and; 2 Department of Microbiology, University of Texas Southwestern Medical Center, Dallas, Texas, USA.

**Keywords:** Immunology, Infectious disease, Microbiology, Autophagy, Innate immunity, Tuberculosis

## Abstract

Autophagy is a critical host defense mechanism that restricts intracellular pathogens such as *Mycobacterium tuberculosis*. A key step in this process is the ubiquitination of *M*. *tuberculosis* or *M*. *tuberculosis*–associated structures. The E3 ligase Smad-specific E3 ubiquitin protein ligase 1 (SMURF1) catalyzes K48-linked ubiquitination, promoting bacterial clearance. However, the function of its homolog, SMURF2, in host defense remains undefined. Here, we demonstrate that *Smurf2* deletion in murine macrophages increases SMURF1 levels, enhances LC3B lipidation, augments K48 ubiquitination of *M*. *tuberculosis*–associated structures, and reduces intracellular *M*. *tuberculosis* replication. These effects are reversed by *Smurf1* deletion, supporting a role for SMURF1 in SMURF2-dependent control of *M*. *tuberculosis*. Mice with myeloid-specific *Smurf2* deletion exhibit modestly prolonged survival after aerosol *M*. *tuberculosis* infection. In human macrophages, *SMURF2* knockdown or its pharmacological inhibition with the HECT E3-ligase inhibitor Heclin reduces *M*. *tuberculosis* replication. Together, our findings identify SMURF2 as a negative regulator of macrophage control of *M*. *tuberculosis* and support further investigation of SMURF2 as a potential target for host-directed therapy in tuberculosis.

## Abstract 2

SMURF2 limits the ability of immune cells to eliminate tuberculosis bacteria, identifying a host pathway that could potentially be targeted to improve infection control.

## Introduction

### M.

*tuberculosis*, the causative agent of tuberculosis (TB), is responsible for more than 10 million new cases of TB and 1.3 million deaths each year, making it the leading cause of death from a single infectious agent worldwide (1). The airborne route of transmission, long latency period, and capacity for immune evasion all contribute to the persistence and global impact of TB. A recent analysis projected over 31 million TB-related deaths between 2020 and 2050, with a corresponding global economic loss of US$17.5 trillion (1).

The standard treatment for TB relies on prolonged multidrug chemotherapy, typically requiring at least 6 months of adherence for drug-susceptible strains. This extended treatment duration, combined with limited diagnostic tools for early detection of drug resistance, has contributed to the emergence of multidrug-resistant and extensively drug-resistant *M*. *tuberculosis* strains (2). These challenges underscore the need for innovative therapeutic strategies that not only shorten treatment but also enhance host immune control of infection. Host-directed therapies aim to bolster the host immune response rather than directly targeting the pathogen, offering the potential to synergize with antibiotics, improve treatment outcomes, and overcome drug resistance (3, 4). However, realizing the promise of host-directed therapy requires a more detailed understanding of host cell-autonomous mechanisms that limit *M*. *tuberculosis* replication.

Autophagy is a conserved intracellular degradation pathway that maintains cellular homeostasis and promotes clearance of intracellular pathogens (5, 6). When the autophagic machinery encounters intracellular pathogens, the ubiquitin-proteasome system is engaged through the attachment of ubiquitin molecules onto intracellular cargo, targeting the cargo for degradation (7–10). Ubiquitinated intracellular cargo is recognized by cargo receptors, such as sequestrosome-1 (SQSTM1/p62), calcium-binding and coiled-coil domain-containing protein 2 (CALCOCO2/NDP52), optineurin, and neighbor of BRCA1 gene 1 (NBR1). These receptors contain both ubiquitin-binding domains and LC3-interacting regions that enable cargo delivery to LC3-decorated autophagosomes for subsequent degradation after lysosomal fusion (10–13).

The ubiquitination cascade is mediated by the sequential action of E1-activating enzymes, E2-conjugating enzymes, and E3 ligases, with E3 ligases conferring substrate specificity (14). A limited number of E3 ligases have been identified that ubiquitinate *M*. *tuberculosis*–containing structures with important roles in xenophagic control of infection (15–17). For example, tripartite motif-containing (TRIM) E3 ligases, such as TRIM32, enhance autophagy in *M*. *tuberculosis*–infected human macrophages (18); PARKIN mediates K63-linked polyubiquitination and recruitment of p62 and NDP52 to *M*. *tuberculosis*–containing structures (19); and Smad-specific E3 ubiquitin protein ligase 1 (SMURF1) promotes K48-linked ubiquitination and proteasome recruitment (10), highlighting distinct and nonredundant mechanisms of bacterial clearance (20).

E3 ligases are regulated by posttranslational modifications such as phosphorylation, acetylation, sumoylation, and ubiquitination (21, 22). In breast cancer cells, SMURF2 negatively regulates SMURF1 by inducing its ubiquitination and degradation (23). SMURF1 and SMURF2 share a conserved structure with an N-terminal C2 domain for membrane binding, WW domains for protein-protein interactions, and a C-terminal HECT (homologous to the E6-AP carboxyl terminus) ubiquitin-ligase domain (24). Notably, human and murine SMURF2 proteins share approximately 97% amino acid sequence identity and possess identical domain architecture, with the catalytic cysteine (C716) within its C-terminal HECT ubiquitin ligase domain at the same position in both species. Initially identified as negative regulators of TGF-β and BMP signaling (25, 26), SMURF1 and SMURF2 have since been implicated in diverse biological processes, including bone metabolism and host-pathogen interactions (10, 27, 28).

Although SMURF1 and SMURF2 share structural similarities and partially overlapping substrates, they also exhibit distinct functions. SMURF2 can induce the degradation of SMURF1, whereas SMURF1 does not affect SMURF2 stability (23). Although both ligases are well studied in cancer biology (29, 30), their roles in cell-autonomous immunity remain poorly understood. Here, we demonstrate that genetic deletion of *Smurf2* in murine macrophages significantly enhances SMURF1 levels, LC3B lipidation, and autophagic targeting of *M*. *tuberculosis*, resulting in reduced bacterial replication. We further show that myeloid-specific *Smurf2* deletion provides modest protection in a murine TB model and that SMURF2 inhibition in human macrophages, both genetically and pharmacologically, enhances bacterial clearance. These findings identify SMURF2 as a negative regulator of cell-autonomous immunity to *M*. *tuberculosis* and a potential target for host-directed therapy in tuberculosis.

## Results

### Smurf2 deletion in BV2 macrophages increases SMURF1 and LC3B-II levels and reduces intracellular M. tuberculosis growth.

To examine the role of SMURF2 in cell-autonomous immunity, we used CRISPR/Cas9 to knock out *Smurf2* in the BV2 mouse microglial cell line. We selected BV2 cells for their suitability in genetic manipulation and their prior use in studies of autophagy and mycobacterial pathogenesis (31–38). SMURF2-deficient BV2 cells showed a 60% increase in SMURF1 protein levels compared with WT controls (Figure 1, A and B). To determine whether SMURF2 regulates intracellular *M*. *tuberculosis* replication, we infected BV2-WT and *Smurf2*-KO cells with *M*. *tuberculosis* and measured bacterial burden by CFU assay. Although *M*. *tuberculosis* grew steadily in control BV2 cells, BV2 *Smurf2*-KO cells exhibited significantly reduced bacterial growth at days 2 and 3 after infection (Figure 1C).

We next examined whether loss of SMURF2 affects autophagy. Lipidation of Atg8 family proteins, including LC3B, is a key step in autophagosome formation (39) and is commonly assessed by monitoring the conversion of LC3B-I to LC3B-II (40, 41). LC3B-II levels were quantified by immunoblot in both BV2- and BV2 *Smurf2*-KO cells under basal conditions and after 4 and 24 hours of *M*. *tuberculosis* infection. There were no differences after 4 hours, but LC3B-II was increased 2-fold in SMURF2-deficient cells at 24 hours after infection (Figure 1, D and E). These findings suggest that SMURF2 negatively regulates autophagy in *M*. *tuberculosis*–infected macrophages, potentially through its effect on SMURF1 stability.

### K48-linked ubiquitin colocalization with M. tuberculosis is increased in BV2 Smurf2-KO macrophages.

SMURF1 promotes K48-linked ubiquitination of substrates, targeting them for proteasomal degradation. We previously showed that loss of SMURF1 reduces K48-linked ubiquitin colocalization with *M*. *tuberculosis* in mouse BMDMs (10). Since SMURF1 levels were elevated in BV2 *Smurf2*-KO cells (Figure 1, A and B), we hypothesized that K48-linked ubiquitination of *M*. *tuberculosis*–associated structures would be correspondingly increased in these cells. To test this, we infected BV2-WT and BV2 *Smurf2*-KO cells with *M*. *tuberculosis* expressing mCherry (*M*. *tuberculosis*–mCherry) and performed immunofluorescence microscopy to assess colocalization with K48-linked ubiquitin. BV2 *Smurf2*-KO cells exhibited a significant increase in K48-linked ubiquitin colocalization with *M*. *tuberculosis*–associated structures compared with WT controls (Figure 2, A and B, and Supplemental Figure 1A; supplemental material available online with this article; https://doi.org/10.1172/jci.insight.199853DS1).

We next evaluated LC3B recruitment to *M*. *tuberculosis*–associated structures under the same conditions. Although LC3B colocalization was modestly increased in BV2 *Smurf2*-KO cells compared with BV2-WT cells, the difference did not reach statistical significance (Figure 2, C and D, and Supplemental Figure 1B). These data indicate that *Smurf2* deficiency enhances K48-linked ubiquitination of *M*. *tuberculosis*–associated structures, consistent with increased SMURF1 expression in BV2 *Smurf2*-KO macrophages.

### Smurf2 deficiency affects M. tuberculosis replication in a SMURF1-dependent manner.

Given that *Smurf2* deletion increased SMURF1 levels and that SMURF1 regulates selective autophagy during *M*. *tuberculosis* infection (10), we tested whether the phenotype observed in BV2 *Smurf2*-KO cells was dependent on SMURF1. We used CRISPR/Cas9 to generate BV2 *Smurf1*-KO and BV2 *Smurf2/1* double-KO cells and quantified SMURF1 and SMURF2 protein levels at baseline. As expected, SMURF1 was increased in BV2 *Smurf2*-KO cells, whereas SMURF2 levels were unchanged in BV2 *Smurf1*-KO cells (Figure 3A).

We next infected BV2-WT, BV2 *Smurf2*-KO, BV2 *Smurf1*-KO, and BV2 *Smurf2/1*-KO cells with *M*. *tuberculosis* and measured intracellular bacterial burden over 3 days. Consistent with prior findings in mouse BMDMs (10), BV2 *Smurf1*-KO cells showed increased *M*. *tuberculosis* replication compared with WT cells. BV2 *Smurf2/1*-KO cells had bacterial burdens comparable to BV2 *Smurf1*-KO cells, indicating that the reduction in *M*. *tuberculosis* growth observed in BV2 *Smurf2*-KO cells requires SMURF1 (Figure 3B).

We also examined LC3B lipidation in these cell lines at baseline and after infection. BV2 *Smurf2*-KO cells showed increased LC3B-II levels, whereas no change in LC3B lipidation was observed in BV2 *Smurf1*-KO or BV2 *Smurf2/1*-KO cells (Figure 3, C and D). This suggests that the effect of *Smurf2* deletion on LC3B lipidation is not mediated by SMURF1. Together, these results indicate that SMURF2 restricts *M*. *tuberculosis* control through SMURF1-dependent mechanisms and modulates LC3B lipidation through a SMURF1-independent pathway.

### SMURF2 complementation restores susceptibility to M. tuberculosis by decreasing SMURF1 protein levels.

SMURF2 contains a conserved catalytic cysteine (C716) within its C-terminal HECT ubiquitin ligase domain, which is essential for its enzymatic activity (Figure 4A) (42). To confirm that the phenotype observed in BV2 *Smurf2*-KO cells was due to loss of SMURF2, we reconstituted SMURF2 expression by lentiviral transduction. In addition, to determine whether SMURF2 suppresses macrophage immunity to *M*. *tuberculosis* through its ligase function, we generated BV2 *Smurf2*-KO macrophages stably complemented with either WT SMURF2 (*Smurf2^WT^*) or a catalytically inactive mutant, C716A (*Smurf2^C716A^*), which targets the active site cysteine. Immunoblot analysis confirmed successful reexpression of SMURF2 in the *Smurf2^WT^*-complemented cells. The catalytically inactive SMURF2^C716A^ mutant was also detected, but its band intensity was significantly reduced compared with the SMURF2^WT^, suggesting lower protein abundance (Figure 4B). We previously observed that SMURF1 expression is increased in the absence of SMURF2 in BV2 cells (Figure 1A and Figure 3A). Consistent with this, complementation with SMURF2^WT^ in BV2 *Smurf2*-KO cells reduced SMURF1 levels. In contrast, the SMURF2^C716A^ mutant failed to reduce SMURF1 protein levels, similar to what was observed in *Smurf2*-KO cells with the empty vector (EV) (Figure 4C). Given the reduced steady-state levels of the SMURF2^C716A^ mutant, we cannot definitively determine whether the failure to restore SMURF1 inhibition by SMURF2 is due to the loss of its catalytic activity or reduced protein abundance. Although these data are consistent with a requirement for SMURF2 E3 ligase activity in regulating SMURF1, the results should be interpreted with caution.

The inability to restore SMURF1 expression in SMURF2^C716A^ mutant–expressing cells may also reflect the lower abundance of the mutant protein.

To test the impact of SMURF2 complementation on *M*. *tuberculosis* replication, we infected the complemented cells with a luminescent *M*. *tuberculosis* strain encoding the entire firefly luciferase operon (*M*. *tuberculosis*–pLux) and quantified luminescence over 3 days. We used the *M*. *tuberculosis*–pLux strain in this and some subsequent experiments when testing multiple conditions simultaneously because the intracellular luminescence of the *M*. *tuberculosis*–pLux strain allows for rapid and continuous readout of *M*. *tuberculosis* burden and comparable results to CFU plating (43, 44). As expected from prior CFU data (Figure 1C and Figure 3B), BV2 *Smurf2*-KO cells transduced with an EV exhibited reduced *M*. *tuberculosis* replication relative to BV2-WT cells. This phenotype was reversed by complementation with *Smurf2^WT^* but not by *Smurf2^C716A^* (Figure 4D). Although we cannot exclude the possibility that the inability of SMURF2^C716A^ to fully restore WT phenotypes reflects reduced protein abundance rather than loss of catalytic function, these findings suggest that the E3 ligase activity of SMURF2 is required to promote intracellular *M*. *tuberculosis* replication in macrophages.

### SMURF2^WT^ but not SMURF2^C716A^ restores K48-linked ubiquitin colocalization with M. tuberculosis and LC3B recruitment to M. tuberculosis–containing structures in BV2 Smurf2-KO macrophages.

To determine whether SMURF2 regulates the recruitment of ubiquitin and autophagy machinery to *M*. *tuberculosis*, we assessed K48-linked ubiquitin and LC3B colocalization with *M*. *tuberculosis*–associated structures in BV2 *Smurf2*-KO cells complemented with either *Smurf2^WT^* or *Smurf2^C716A^*. BV2 *Smurf2*-KO cells transduced with an EV exhibited increased K48-linked ubiquitin colocalization with *M*. *tuberculosis*–associated structures (Figure 5, A and B, and Supplemental Figure 1C), consistent with earlier observations (Figure 2A). This phenotype was reversed by complementation with *Smurf2^WT^*, which restored K48-linked ubiquitin signal to levels observed in BV2-WT cells. In contrast, complementation with the catalytically inactive *Smurf2^C716A^* mutant failed to reduce K48 ubiquitin colocalization, indicating that SMURF2 ligase activity is required for this function (Figure 5, A and B, and Supplemental Figure 1C).

We next examined LC3B recruitment to *M*. *tuberculosis*–associated structures. A modest increase in LC3B colocalization was again observed in BV2 *Smurf2*-KO cells relative to WT, although the difference did not reach statistical significance (Figure 5, C and D, and Supplemental Figure 1D). Complementation with *Smurf2^WT^* significantly reduced LC3B colocalization, restoring levels to those observed in BV2-WT (EV) cells. In contrast, complementation with the *Smurf2^C716A^* mutant failed to reduce LC3B colocalization.

These results demonstrate that SMURF2 regulates the accumulation of K48-linked ubiquitin at *M*. *tuberculosis*–associated structures in a manner that depends on its catalytic activity, although differences in protein expression between SMURF2^WT^ and SMURF2^C716A^ may also contribute.

### Primary mouse BMDMs lacking Smurf2 exhibit increased resistance to M. tuberculosis infection.

To investigate the role of SMURF2 in primary macrophages, we generated mice with myeloid-specific deletion of *Smurf2* by crossing *Smurf2^fl/fl^* mice (45) with *LysMCre* transgenic mice (46). Western blot analysis of BMDMs confirmed recombination at the *Smurf2* locus, with an average 84% reduction in SMURF2 protein levels in *LysMCre*^+/–^*Smurf2^fl/fl^* BMDMs compared with littermate controls (Supplemental Figure 2).

To assess functional consequences of SMURF2 depletion, we infected *Smurf2^fl/fl^* and *LysMCre*^+/–^*Smurf2^fl/fl^* BMDMs with *M*. *tuberculosis* and quantified bacterial burden over a 3-day time course. Like BV2 *Smurf2*-KO cells, *LysMCre*^+/–^*Smurf2^fl/fl^* BMDMs showed reduced intracellular *M*. *tuberculosis* replication compared with controls at day 3 after infection (Figure 6A). We next examined additional molecular consequences of SMURF2 depletion. Immunoblot analysis confirmed reduced SMURF2 expression in *LysMCre*^+/–^*Smurf2^fl/fl^* BMDMs after infection (Figure 6, B and C). However, in contrast to BV2 *Smurf2*-KO cells, although SMURF1 protein levels were modestly increased in *LysMCre*^+/–^*Smurf2^fl/fl^* BMDMs compared with controls after *M*. *tuberculosis* infection, these results were not statistically significant (Figure 6, B and D). LC3B lipidation was also not significantly different between genotypes (Figure 6, B and E).

To further assess autophagic targeting of *M*. *tuberculosis*, we performed immunofluorescence analysis of LC3B colocalization with *M*. *tuberculosis*–mCherry. As in BV2 cells, *LysMCre*^+/–^ *Smurf2^fl/fl^* BMDMs exhibited a modest increase in LC3B recruitment to *M*. *tuberculosis*–associated structures, but the difference was not statistically significant (Figure 6, F and G, and Supplemental Figure 1E).

These results indicate that partial depletion of SMURF2 in primary BMDMs is sufficient to reduce *M*. *tuberculosis* replication, although the associated changes in SMURF1 expression and LC3B lipidation are less pronounced than in BV2 *Smurf2*-KO cells. Residual SMURF2 expression in *LysMCre^+/–^Smurf2^fl/fl^* BMDMs compared with BV2 *Smurf2*-KO cells may contribute to the more modest phenotype.

### Smurf2 does not affect bacterial burden or lung pathology during M. tuberculosis infection in vivo.

We next tested whether *Smurf2* contributes to host defense against *M*. *tuberculosis* infection in vivo. *LysMCre*^+/–^*Smurf2^fl/fl^* and littermate *Smurf2^fl/fl^* control mice were infected via aerosol with a low-dose inoculum of *M*. *tuberculosis* (~50 CFU) (Figure 7A) (47–49). At 7, 21, and 42 days after infection, we quantified bacterial burden in the lungs, spleen, and liver. No significant differences in CFU were observed at any time point between groups (Figure 7, B–D). To assess the impact of *Smurf2* deletion on tissue pathology, lung sections were examined by histology at days 21 and 42 after infection. *LysMCre*^+/–^*Smurf2^fl/fl^* mice showed similar degrees of inflammation compared with *Smurf2^fl/fl^* controls (Figure 7, E and F).

We then monitored survival after aerosol infection. When male and female mice were analyzed together, survival curves were not significantly different (Figure 7G). However, sex-stratified analysis revealed that female *LysMCre*^+/–^*Smurf2^fl/fl^* mice survived significantly longer than female *Smurf2^fl/fl^* littermates, whereas no survival difference was observed among males (Figure 7, H and I).

To determine whether the increased survival observed in *LysMCre*^+/–^*Smurf2^fl/fl^* females was driven by sex-specific differences in *Smurf2* deletion efficiency, we quantified mRNA and protein levels in BMDMs from both sexes. RT-qPCR and Western blot analyses confirmed robust reduction of mRNA and protein, with no significant differences in deletion efficiency between males and females (Supplemental Figure 3). These findings suggest that the sex-specific survival rate observed in vivo is not a result of differential *Smurf2* deletion efficiency but may instead reflect broader systemic immunological or hormonal differences.

### SMURF2 knockdown reduces M. tuberculosis intracellular replication in human macrophages.

To determine whether SMURF2 contributes to *M*. *tuberculosis* control in human cells, we first differentiated the human monocytic cell lines THP-1 and U937 into macrophages.

Prior to differentiation, these cells were transduced with lentiviral vectors encoding a nontargeting control shRNA or a shRNA targeting *SMURF2*. We observed a significant reduction in *SMURF2* mRNA expression in both cell lines (Figure 8, A and C). We also infected differentiated THP-1 and U937 cells with *M*. *tuberculosis*–pLux and measured luminescence over 3 days, and we found that the knockdown of *SMURF2* significantly restricted intracellular bacterial growth in human cells compared with the nontargeting shRNA controls (Figure 8, B and D).

To confirm these findings in a more physiologically relevant cell model, we differentiated primary human monocyte-derived macrophages (hMDMs) from CD14^+^-monocytes isolated from healthy donors. After transduction with lentiviral vectors encoding a nontargeting control shRNA or 1 of 2 independent shRNAs targeting *SMURF2* (Figure 8E), knockdown efficiency was analyzed by immunoblot. We observed varying degrees of SMURF2 depletion across the 2 *SMURF2* shRNA constructs (Figure 8F). We then infected hMDMs with *M*. *tuberculosis* and quantified intracellular bacterial burden over a 3-day time course by CFU analysis. Both *SMURF2*-targeting shRNAs significantly reduced *M*. *tuberculosis* replication compared with the nontargeting control (Figure 8G). Notably, cells expressing the shRNA that achieved more efficient *SMURF2* knockdown (shRNA 2) also demonstrated a lower bacterial burden, suggesting a dose-dependent relationship between SMURF2 expression and *M*. *tuberculosis* growth in hMDMs. These data demonstrate that SMURF2 negatively regulates macrophage control of *M*. *tuberculosis* in multiple human macrophage systems, including THP-1 cells, U937 cells, and primary human macrophages, supporting a conserved role for SMURF2 in suppressing cell-autonomous immunity across species.

### Pharmacological inhibition of SMURF2 reduces M. tuberculosis replication and enhances LC3B recruitment in human macrophages.

To evaluate whether pharmacological inhibition of SMURF2 could enhance host control of *M*. *tuberculosis*, we treated hMDMs with Heclin, a small molecule that targets multiple HECT-type E3 ligases including SMURF2 (Supplemental Figure 4A) (50). We infected hMDMs with *M*. *tuberculosis*–pLux, treated cells with increasing concentrations of Heclin, and measured luminescence over 3 days. Heclin reduced intracellular *M*. *tuberculosis* replication in a dose-dependent manner at all time points (Figure 9A). To determine whether Heclin directly affects *M*. *tuberculosis* viability, axenic *M*. *tuberculosis* cultures were incubated with Heclin or DMSO, and growth was measured by optical density. Heclin alone had no impact on axenic *M*. *tuberculosis* growth, confirming that the reduction in luminescence observed in macrophages was not due to a direct antimicrobial effect (Supplemental Figure 4B).

Since Heclin inhibits several HECT ligases, including SMURF2 (IC_50_ 6.8 μM), NEDD4 (IC_50_ 6.3 μM), and WWP1 (IC_50_ 6.9 μM) (50), we sought to determine whether its antibacterial effect in macrophages was mediated specifically through SMURF2. To do this, we infected both BV2-WT and BV2 *Smurf2*-KO cells with *M*. *tuberculosis*–pLux and treated them with increasing concentrations of Heclin. Like hMDMs, BV2-WT cells exhibited a dose-dependent reduction in *M*. *tuberculosis* replication, whereas BV2 *Smurf2*-KO cells were unresponsive to Heclin treatment but showed reduced bacterial levels similar to the highest tested Heclin concentration (Figure 9B). These results indicate that the *M*. *tuberculosis*–restrictive effect of Heclin in macrophages is dependent on SMURF2.

To assess the effect of SMURF2 inhibition on autophagy, we quantified LC3B colocalization with *M*. *tuberculosis*–associated structures in Heclin- or vehicle-treated hMDMs. Heclin treatment significantly increased LC3B recruitment to *M*. *tuberculosis*–mCherry in hMDMs, consistent with enhanced selective autophagy (Figure 9, C and D, and Supplemental Figure 1E). We also attempted to assess K48-linked ubiquitin colocalization, but the K48-ubiquitin staining was inconsistent and could not be reliably quantified.

Together, these findings support a role for SMURF2 as a negative regulator of selective autophagy in human macrophages and demonstrate that its pharmacological inhibition enhances both murine and human macrophage control of *M*. *tuberculosis*.

## Discussion

Our findings identify SMURF2 as a negative regulator of autophagy-mediated, cell-autonomous immunity to *M*. *tuberculosis*. In murine macrophages, *Smurf2* deletion increased SMURF1 abundance, enhanced K48-linked ubiquitination, and reduced bacterial replication. We also observed a trend toward increased LC3B recruitment to *M*. *tuberculosis*–associated structures in murine macrophages lacking SMURF2. These effects were lost in the absence of SMURF1, consistent with prior evidence that SMURF2 promotes SMURF1 degradation (23) and with a model in which SMURF2 limits autophagy, at least in part, through regulation of SMURF1 stability. We extended these observations to primary human macrophages, where both genetic and pharmacological inhibition of SMURF2 increased LC3B recruitment and reduced *M*. *tuberculosis* growth without direct antibacterial effects on axenic growth. The concordance between murine and human data supports a conserved role for SMURF2 in macrophage control of *M*. *tuberculosis* and suggests that SMURF2 warrants further investigation as a potential target for host-directed therapy.

Although our current findings highlight the role of the SMURF2/SMURF1 axis on K48-linked ubiquitination of *M*. *tuberculosis*, the role of alternative ubiquitin linkages, such as K63-linked chains, remains a critical aspect of selective autophagy. Previous work has shown that the E3 ligase PARKIN promotes host resistance to *M*. *tuberculosis* by catalyzing the K63-linked ubiquitination of bacteria (19). It remains to be determined whether SMURF2 concomitantly modulates K63-linked ubiquitin recruitment to *M*. *tuberculosis*. Investigating this interaction represents an important future direction for clarifying the mechanisms by which SMURF2 regulates cell-autonomous immunity. Furthermore, although our study focuses on selective autophagy, we acknowledge that additional antimicrobial mechanisms, such as host-mediated oxidative stress, may also contribute to the observed SMURF2-associated phenotype. Describing the contributions of these parallel pathways remains to be investigated.

E3 ubiquitin ligases, including SMURF2, are central regulators of protein ubiquitination, influencing diverse immune processes such as lymphocyte development, antigen presentation, and immune evasion (51–53). Although their antiviral functions are well described (27, 54, 55), the role of HECT E3 ligases in antibacterial immunity is less understood. Several ligases, including TRIM32, NEDD4, and SMURF1, promote autophagy-mediated *M*. *tuberculosis* clearance by enhancing bacterial ubiquitination or stabilizing key autophagy proteins such as BECN1 (18, 56). Other ligases, such as TRIM27, promote bacterial clearance by activating transcription factors like TFEB, independent of their ligase activity (57).

### M.

*tuberculosis* can also subvert host E3 ligases to favor its survival. For example, the *M*. *tuberculosis* PE/PPE protein PE5 hijacks the CRL2 complex to suppress cell-autonomous immunity and promote its intracellular replication (58), and *M*. *tuberculosis* Rv0222 interacts with ANAPC2 to dampen proinflammatory cytokine production (59). Finally, PPE36 promotes SMURF1-dependent ubiquitination and proteasomal degradation of MyD88, limiting host inflammatory responses (60).

Our initial findings demonstrate the importance of SMURF2 in mediating host-pathogen interactions. However, it is essential to address the experimental nuances inherent in our models. Immortalized BV2 cells and primary macrophages differ in their baseline proliferative state, metabolic activity, and autophagic tone (61). These physiological differences likely contribute to the enhanced LC3B- and K48-linked ubiquitin phenotypes observed in experiments performed in BV2 cells. Importantly, enhanced restriction of *M*. *tuberculosis* intracellular growth after SMURF2 perturbation was consistently observed across multiple murine and human macrophage systems, including BV2 cells, BMDMs, THP-1 cells, U937 cells, and primary human macrophages. This reproducibility across species and cellular contexts highlights the functional importance of SMURF2 during *M*. *tuberculosis* infection. Furthermore, the discrepancy between intracellular bacterial restriction and LC3B lipidation and recruitment in BMDMs suggests that the SMURF2-dependent effects are not mediated exclusively through canonical selective autophagy pathways in murine primary macrophages.

SMURF2 deficiency enhanced bacterial clearance in vitro, but the in vivo effects were more modest: overall bacterial burden and lung inflammation were unchanged, but female *LysMCre^+/–^Smurf2^fl/fl^* mice exhibited prolonged survival compared with littermate controls. Mice were infected using a low-dose aerosol challenge (~50 CFU) to better reflect physiological exposure in humans. Although core autophagy gene knockouts are correlated with clear susceptibility phenotypes under low-dose aerosol infection conditions (62), the modest phenotype observed here likely reflects the specific role of SMURF2, incomplete deletion within relevant myeloid populations, compensatory immune mechanisms, or a combination of these factors. Furthermore, our in vitro data indicate that SMURF2 regulates SMURF1 protein stability, but direct biochemical assessment of SMURF1 levels in infected lung macrophages in vivo remains challenging due to the limited number of infected cells recoverable from low-dose aerosol infection models.

Our results also suggest sex-specific differences in survival rates observed in vivo, a phenotype that is in accordance with known sex-based differences in TB susceptibility (49, 63–65) and autophagy regulation (66). Whether this phenotype reflects immunological and/or hormonal differences that are independent of deletion efficiency itself, it warrants further study. We note that our original in vivo experiments were not designed for sex-stratified analysis of bacterial burden and pathology at different time points; consequently, these measurements should be interpreted with caution regarding potential sex-specific differences.

Our experiments in human macrophages underscore the translational importance of these findings, as SMURF2 knockdown or pharmacological inhibition with the HECT E3 ligase Heclin significantly reduced *M*. *tuberculosis* replication without direct antibacterial effects and increased LC3B colocalization with *M*. *tuberculosis*. However, we acknowledge limitations related to this pharmacological approach. Heclin inhibits multiple HECT E3 ligases, including NEDD4 and WWP1, and therefore cannot be considered a selective inhibitor of SMURF2. Nevertheless, the absence of an effect of Heclin in *Smurf2*-deficient cells indicates that its *M*. *tuberculosis*–restrictive activity in macrophages is dependent on SMURF2. Potential effects of Heclin on other HECT E3 ligases, as well as potential cellular toxicity, warrant caution in interpreting these pharmacological studies and highlight the need for more selective SMURF2 inhibitors. Furthermore, despite several attempts using commercially available antibodies, we were unable to obtain robust, reproducible signal-to-noise ratios to support reliable quantitative K48-linked ubiquitin/*M*. *tuberculosis*–mCherry colocalization analysis in hMDMs. Despite these limitations, the conserved bacterial restriction phenotype observed across hMDMs, THP-1 cells, and U937 cells using both pharmacological and genetic approaches supports a role for SMURF2 in macrophage control of *M*. *tuberculosis*.

Although autophagy has been widely studied as a cell-autonomous defense against intracellular pathogens, including *M*. *tuberculosis*, there is a lack of consensus from in vivo models regarding its protective function (62, 67–69). Conflicting results from murine models may reflect differences in the *M*. *tuberculosis* strain used, infectious dose, mouse background, specificity of gene deletion, and duration of survival studies. Moreover, *M*. *tuberculosis* encodes virulence factors that modulate autophagy or promote autophagy evasion. For example, the secreted effector enhanced intracellular survival (Eis) inhibits autophagy initiation through modulation of host redox-dependent signaling and acetylation pathways (70, 71), and PE_PGRS47 inhibits autophagy initiation by interacting with Ras-related protein Rab1a (72, 73). Likewise, the SecA2-dependent protein export system exports effectors, including protein kinase G and secreted acid phosphatase, to block phagosome-lysosome fusion through blockade of the small GTPase Rab7 (74, 75). *M*. *tuberculosis* lipids, including phthiocerol dimycocerosates and sulfolipid-1, further impair phagosome maturation and autophagic targeting (76–79). These strategies underscore the importance of autophagy as a barrier to infection and suggest that *M*. *tuberculosis* has evolved multiple mechanisms to subvert it.

In contrast to the debated role of autophagy in TB pathogenesis in mice, evidence from human macrophages consistently supports its contribution to cell-autonomous defense. Genetic ablation of *SMURF1* (10), *IRGM* (80), *ATG7* (81), or *ATG14* (81) in human macrophages increases mycobacterial replication, whereas inhibition of the deubiquitinase USP15, which counters PARKIN, decreases *M*. *tuberculosis* replication (82). Our findings add to this body of evidence by showing that both genetic and pharmacological disruption of SMURF2 enhances *M*. *tuberculosis* clearance in primary human macrophages by stimulating autophagy. In addition, recent work demonstrates that calcium leakage after *M*. *tuberculosis* phagosome damage induces multimeric ATG8/LC3 lipidation, limiting bacterial replication through a noncanonical autophagy process (83). Together, these findings underscore that selective autophagy, through both canonical and noncanonical mechanisms, is a key component of human host defense against *M*. *tuberculosis* and a promising target for therapeutic intervention.

In summary, our data define SMURF2 as a negative regulator of autophagy-mediated control of *M*. *tuberculosis* and provide mechanistic insight into how E3 ligases shape cell-autonomous immunity. These findings support further exploration of SMURF2 as a therapeutic target for TB, particularly in combination with standard antimicrobial regimens.

## Methods

### Sex as a biological variable.

For murine BMDM studies, our study examined male and female animals, and findings are reported for both sexes. For hMDM studies, we isolated monocytes from blood obtained from anonymous donors acquired from a local blood bank; therefore, information about donor sex is unavailable.

### Mice.

To obtain *LysMCre^+/–^Smurf2^fl/fl^* mice, we crossed *LysMCre mice* (strain 004781, The Jackson Laboratory) (46) with *Smurf2^fl/fl^* mice (a gift from Ying E. Zhang, The National Cancer Institute Center for Cancer Research, Bethesda, Maryland, USA) (45). Genotyping of mice was performed according to the protocols provided for each strain on The Jackson Laboratory website. Briefly, genomic DNA was extracted from ear punches using a previously described protocol (84). All mice were maintained in a pathogen-free facility with a 12-hour light/12-hour dark cycle and fed ad libitum in accordance with institutional guidelines.

### Bacterial strains.

We used the Erdman strain of *M*. *tuberculosis* as the parental strain for this study. The *M*. *tuberculosis* mCherry-expressing strain has been described previously (10, 47, 85). The Erdman luminescent reporter strain was generated by transforming the parental *M*. *tuberculosis* strain with pMV306hsp+LuxG13 plasmid (a gift from Brian Robertson and Siouxsie Wiles, Addgene plasmid 26161) (86). *M*. *tuberculosis* cultures were grown to mid-log phase in Middlebrook 7H9 medium (Difco), supplemented with 10% Middlebrook OADC (BD Biosciences), 0.5% glycerol, and 0.05% Tween-80 at 37°C using aseptic techniques in a biosafety level 3 laboratory. Strains were propagated with minimal passage to maintain virulence. After growth of *M*. *tuberculosis*, bacteria were washed with PBS and sonicated with 3 pulses lasting 7 seconds each, with an amplitude of 90% and 5-second rests between pulses. After sonication, the concentration of bacterial suspensions was determined by measuring OD_600_, using the formula: 1 OD_600_ = 3 × 10^8^ bacteria/mL, and diluted in macrophage media prior to infection.

To assess the effect of the HECT E3 ligase inhibitor Heclin on *M*. *tuberculosis* growth, Erdman strain cultures were grown in 7H9 medium supplemented with 10% OADC, 0.5% glycerol, and 0.05% Tween-80 in the presence of either 10 μM Heclin or 0.1% DMSO (vehicle control). Bacterial growth was monitored by measuring OD_600_ at different time points.

### Cell culture.

The BV2 mouse microglial cells (WT and Smurf2 CRISPR/Cas9-based KO) were provided by the Genome Engineering and iPSC Center at Washington University in St. Louis, Missouri, USA. BV2 cells were cultured in complete DMEM supplemented with 10% FBS (Gibco) and 10 mM HEPES (Thermo Fisher Scientific) and maintained in 5% CO_2_ at 37°C. BMDMs were isolated and cultured from 8–12-week-old mice femurs and tibia as previously described (47).

The human monocytic cell lines THP-1 (TIB-202) and U937 (CRL-1593.2) were obtained from ATCC and cultured in RPMI 1640 medium supplemented with 10% FBS (Gibco), 10 mM HEPES (Thermo Fisher Scientific), 2 mM L-glutamine, and 1 mM sodium pyruvate (Gibco). For *SMURF2* knockdown cells, puromycin was added to maintain selection pressure at final concentrations of 2 μg/mL for THP-1 and 1 μg/mL for U937 cells. To induce differentiation into macrophage cells, THP-1 and U937 cells were treated with 100 ng/mL PMA (Sigma-Aldrich, P8139) for 48 hours. After incubation, the medium was replaced by fresh, PMA-free complete RPMI to allow for cell recovery before experimental procedures.

PBMCs were isolated from buffy coats of healthy donors using SepMate tubes (StemCell Technologies) and enriched for CD14^+^ monocytes using MACS CD14^+^ microbeads (Miltenyi Biotec), following the manufacturer’s protocol. Adherent human macrophages (hMDMs) were obtained by culturing CD14^+^ monocytes in complete RPMI 1640 (supplemented with 10% FBS, 2 mM L-glutamine, 100 U/mL penicillin, 100 μg/mL streptomycin), with 10% inactivated human serum and 50 ng/mL recombinant human M-CSF (R&D Systems) for 7 days. Lentiviral transductions for *SMURF2* knockdown were performed using the protocol described below.

### Lentiviral transduction for Smurf2 overexpression in BV2 cells.

To achieve transgenic expression of SMURF2 in BV2 cells, we cloned full-length *Smurf2* cDNA (*Smurf2^WT^* or catalytic-dead *Smurf2^C716A^*) (87) into the entry vector pENTR/D-TOPO, which contains a Myc-tag sequence. These constructs were then subcloned into the lentiviral GATEWAY destination vector pLENTI CMV Blast using the LR Clonase II enzyme mix (Thermo Fisher Scientific). All plasmids were sequenced to verify correct gene insertion. Lentiviral particles were generated by transfecting HEK293T cells with the lentiviral constructs along with the packaging vectors pMD2.G and psPAX2 using FuGene HD transfection reagent (Promega), following the manufacturer’s protocol. After 48 hours of incubation in 5% CO_2_ at 37°C, virus-containing supernatants were collected, filtered through a 0.45 μm filter (MilliporeSigma), and added to BV2 cell cultures in the presence of 8 μg/mL polybrene (MilliporeSigma). Cells were incubated in 5% CO_2_ at 37°C for 16 hours, when the medium was replaced with fresh complete DMEM containing 4 μg/mL blasticidin. The medium was changed every other day until resistant cell populations were established.

### Lentiviral transduction for SMURF2 knockdown in human macrophages.

shRNA synthetic molecules used to induce sequence-specific *SMURF2* knockdown in hMDMs (clone TRCN0000003477, target sequence: CGCCTCAAAGACACTGGTTAT; or clone TRCN0000003478, target sequence: CCACCCTATGAAAGCTATGAA) were obtained from Sigma-Aldrich, with a nontargeting sequence serving as a control. Lentiviral particles containing shRNAs were produced in HEK293T cells as described previously.

For THP-1 and U937 cell lines, cells were transduced with lentiviral particles expressing clone TRCN0000003477. After the establishment of puromycin-resistant populations, cells were differentiated with PMA, as described previously.

For hMDMs, cells were transduced on day 4 of differentiation by adding lentivirus-containing supernatants to the cultures in the presence of 8 μg/mL polybrene (MilliporeSigma). Cells were transduced via spinoculation at 800*g* for 1 hour at 30°C. After transduction, the medium was replaced with fresh complete RPMI 1640 supplemented with 50 ng/mL GM-CSF. On day 5, the medium was replaced with GM-CSF–free RPMI 1640 containing 2 μg/mL puromycin for selection. On day 7, MDMs were infected with *M*. *tuberculosis*, as described below.

Knockdown efficiency was calculated as the percentage decrease in the SMURF2/GAPDH ratio in *SMURF2* shRNA–transduced cells relative to nontargeting shRNA–transduced cells.

### Intracellular quantification of M. tuberculosis.

To enumerate the number of intracellular bacteria by the CFU method, we infected macrophages with *M*. *tuberculosis* at an MOI of 1. After phagocytosis, cells were washed twice with PBS and lysed at different time points with 0.5% Triton X-100 in PBS. Serial dilutions were plated on 7H11 plates supplemented with 10% OADC, 0.5% glycerol, and 50 μg/mL cycloheximide, and grown for 3 to 4 weeks at 37°C.

To monitor intracellular mycobacterial growth by luminescence, we infected macrophages with the luminescent reporter strain at an MOI of 3, and after phagocytosis, cells were washed twice with PBS, and fresh media was added to each well prior to the measurement. Luminescence was measured using a BioTek Synergy Neo2 Hybrid Multimode Reader every 24 hours for 3 days.

To assess the effect of the HECT E3 ligase inhibitor Heclin (Sigma-Aldrich) on intracellular *M*. *tuberculosis* quantification, cells were treated with different concentrations of the inhibitor (diluted in DMSO) after phagocytosis and before cell lysis for Western blot or bacterial growth in macrophages.

### Western blot.

Cells were seeded at 5 × 10^5^ cells per well in 12-well plates and maintained at 37°C with 5% CO_2_ for 4 hours prior to infection with Erdman *M*. *tuberculosis* at an MOI of 5. After phagocytosis, cells were maintained in complete DMEM with or without 0.1% DMSO or 10 μM Heclin. At different time points, cells were washed twice with PBS and lysed in RIPA buffer (Cell Signaling Technology) supplemented with cOmplete EDTA-free protease inhibitor cocktail (Roche) and Halt phosphatase inhibitor cocktail (Thermo Fisher Scientific). Lysates were incubated on ice for 20 minutes, and then collected and filtered twice through a 0.2 μm filter (MilliporeSigma). Laemmli sample buffer was added, and samples were boiled for 3 minutes. Proteins were separated by SDS-PAGE using 4%–20% polyacrylamide resolving gel (Bio-Rad). After electrophoresis, proteins were transferred to PVDF membranes and blocked with 5% BSA in Tris-buffered saline with 0.1% Tween-20 (TBS-T). To detect SMURF2, SMURF1, and LC3B, the following antibodies were used: anti-LC3B (Novus, NB100-2220), SMURF2 (Cell Signaling Technology, S12024), and SMURF1 (Santa Cruz Biotechnology, clone 45-K, sc-100616). Anti-β-ACTIN (Santa Cruz Biotechnology, clone C4, sc-47778) was used as a loading control. Membranes were developed using Clarity Western ECL Substrate (Bio-Rad).

### Immunofluorescence analysis.

Cells were seeded at 1 × 10^5^ cells per well in 24-well plates with glass coverslips and maintained at 37°C with 5% CO_2_ for 4 hours prior to infection with mCherry-expressing *M*. *tuberculosis* at an MOI of 1 for 17 hours. Coverslips were then washed twice with PBS, fixed in 1% paraformaldehyde for 16 hours at 4°C, and washed twice again in PBS. For K48 ubiquitin staining, coverslips were permeabilized with 0.3% Triton X-100 in PBS (PBS-T) for 15 minutes, blocked with 3% BSA in PBS-T for 1 hour, and incubated with humanized anti-K48 ubiquitin antibody (clone Apu2.07, developed by Genentech) for 1 hour at room temperature. Coverslips were then washed 3 times with PBS and incubated with Alexa Fluor 488–conjugated goat anti-human IgG (Thermo Fisher Scientific, A-11013) for 1 hour at room temperature, protected from light. For LC3B staining, coverslips were permeabilized with 100% methanol for 6 minutes at –20°C, blocked with 3% BSA in PBS for 1 hour, and incubated with rabbit anti-LC3B (Novus) for 1 hour at room temperature. Coverslips were then washed 3 times with PBS and incubated with Alexa Fluor 488–conjugated goat anti-rabbit IgG (Thermo Fisher Scientific, A-11008) for 1 hour at room temperature, protected from light. After 3 additional washes with PBS, all coverslips were mounted using ProLong Diamond Antifade Mountant with DAPI and sealed with nail polish. Slides were visualized using a Nikon CSU-W1 SoRa spinning disk confocal microscope with a 60× objective. Colocalization event counting was performed in a blinded manner, with samples numbered to prevent investigator bias.

### Low-dose aerosol infection of mice with M.

*tuberculosis*. *Smurf2^fl/fl^* and *LysMCre^+/–^Smurf2^fl/fl^* mice were infected via aerosol as previously described (47, 62). Briefly, parental *M*. *tuberculosis* cultures grown to mid-log phase were washed 3 times with PBS, sonicated as described above, and resuspended in PBS to an OD_600_ of 0.1 in PBS. Bacterial suspensions were used to infect mice in an aerosol chamber (GlasCol Inc.), with an inoculum of approximately 50 bacteria per mouse. On day 0, both lungs from 5 mice were plated to determine the initial bacterial load. At 7, 21, and 42 days after infection, lungs, liver, and spleen were collected; homogenized in PBS; and plated on 7H11 agar plates for CFU enumeration. For survival studies, mice were weighed weekly and euthanized upon reaching 15% loss of their maximum body weight, a threshold marker of imminent death, as has been previously reported (47).

### Histological analysis.

The left lung lobe from each mouse was collected at indicated time points and fixed in 10% formaldehyde in PBS, pH 7.2. Tissues were then transferred to 70% ethanol and submitted to the Molecular Pathology Core Facility at University of Texas Southwestern (UTSW) for paraffin embedding, sectioning (5 μm thickness), and H&E staining. Slides were scanned at 20× magnification using a NanoZoomer S60 digital slide scanner. The percentage of inflammation per tissue was subsequently calculated using ImageJ (NIH) by observers blinded to mouse genotype.

### Statistics.

All statistical analysis was conducted using GraphPad Prism software (version 10). For in vitro studies, data were analyzed using an unpaired, 2-tailed *t* test for comparisons between 2 groups and 2-way ANOVA for comparisons with more than 2 groups. For in vivo studies, the Mann-Whitney *U* test was used for nonparametric comparisons between 2 groups of mice. For mouse mortality studies, Kaplan-Meier survival curves were generated and analyzed through log-rank tests to determine statistical significance. Densitometric analysis of immunoblots was performed using ImageJ (NIH) (version 1.54f). The specific statistical tests used are indicated in the figure legends. *P* values of 0.05 or less were considered statistically significant.

### Study approval.

All animal experiments were approved by the IACUC at UTSW Medical Center and were conducted in accordance with the NIH *Guide for the Care and Use of Laboratory Animals* (National Academies Press, 2011). UTSW is accredited by the American Association for Accreditation of Laboratory Animal Care (AAALAC).

### Data availability.

Values for all data points in graphs are reported in the Supporting Data Values file. Additional data and detailed information are available from the corresponding author upon request. No custom analytic code was generated for this study.

## Author contributions

PCC and MUS conceptualized the study, conducted the formal analysis, and wrote the original draft. MUS acquired funding, administered the project, and supervised the study. PCC, KCR, VAE, BRSD, KFN, and SAA conducted the investigation. PCC performed visualization. All authors reviewed and edited the manuscript.

## Conflict of interest

The authors have declared that no conflict of interest exists.

## Funding support

This work is the result of NIH funding, in whole or in part, and is subject to the NIH Public Access Policy. Through acceptance of this federal funding, the NIH has been given a right to make the work publicly available in PubMed Central.

- NIH grant U19 AI142784 (to MUS).
- NIH grant R01 AI187291 (to MUS).
- NIH grant T32 HL098040 (to KCR).
- NIH grant T32 AI007520 (to KFN).

## Supplementary Material

Supplemental data

Unedited blot and gel images

Supporting data values

## Figures and Tables

**Figure 1:**
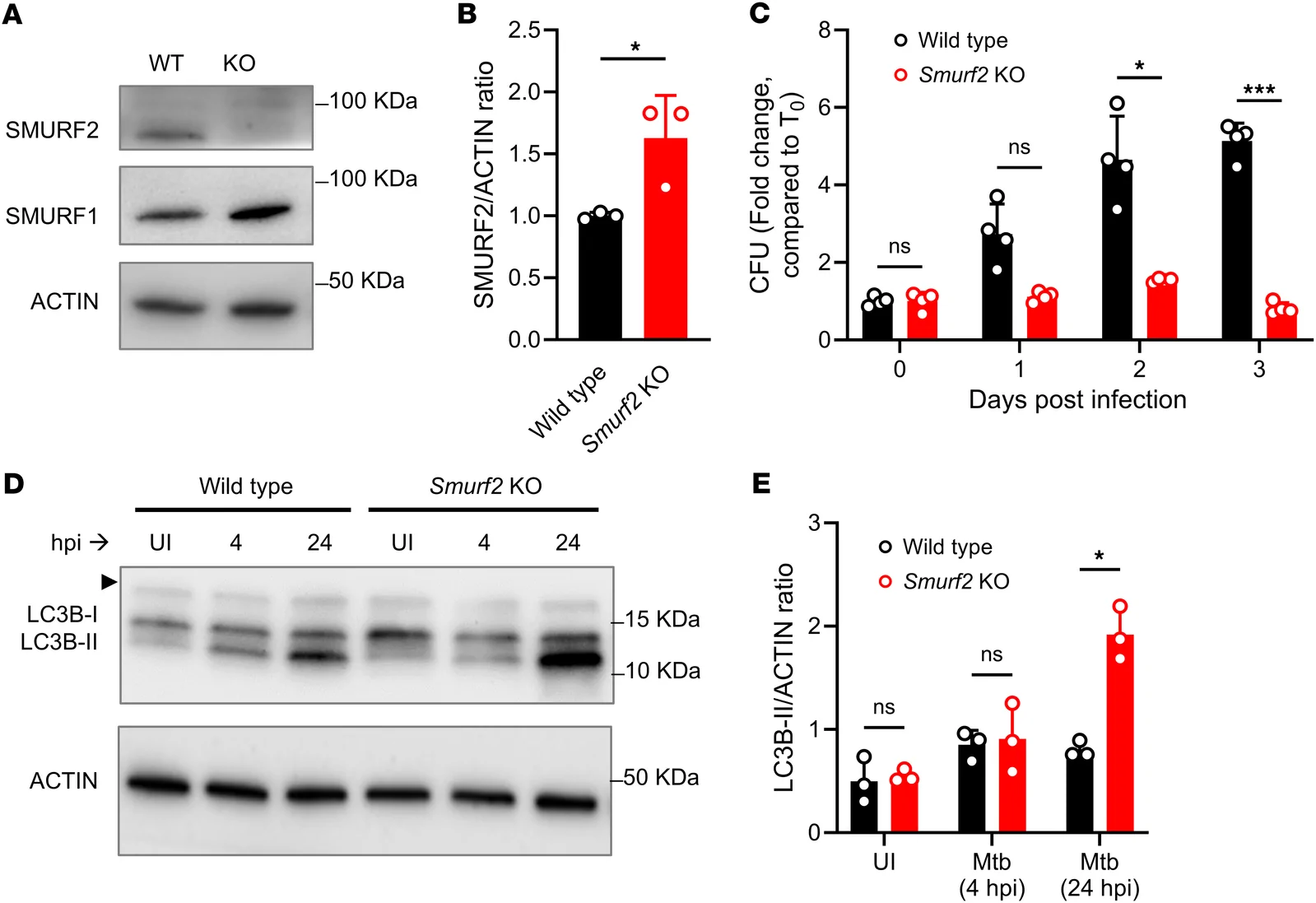
Murine macrophages lacking *Smurf2* are more resistant to *M*. *tuberculosis* and show an increased LC3B lipidation. (**A**) Representative immunoblot analysis of SMURF2 and SMURF1 accumulation in BV2-WT and BV2 *Smurf2*-KO cells under basal conditions. (**B**) Densitometric quantification of SMURF1 levels, as shown in **A** (mean ± SD of 3 independent experiments, *P ≤ 0.05; unpaired *t* test). (**C**) Intracellular *M*. *tuberculosis* CFU in BV2-WT and *Smurf2*-KO cells at different time points after infection (mean ± SD). Data are representative of 3 independent experiments performed in quadruplicate (ns, nonsignificant, *P ≤ 0.05 and ***P ≤ 0.001; 2-way ANOVA). (**D**) Representative immunoblot of LC3B-II levels in BV2-WT and *Smurf2*-KO cells under basal (UI) and at 4 and 24 hours after infection (hpi). Arrowhead indicates unspecific binding of the LC3B antibody. (**E**) Densitometric quantification of LC3B-II levels, as shown in **D** (mean ± SD of 3 independent experiments, ns, nonsignificant, *P ≤ 0.05; 2-way ANOVA).

**Figure 2:**
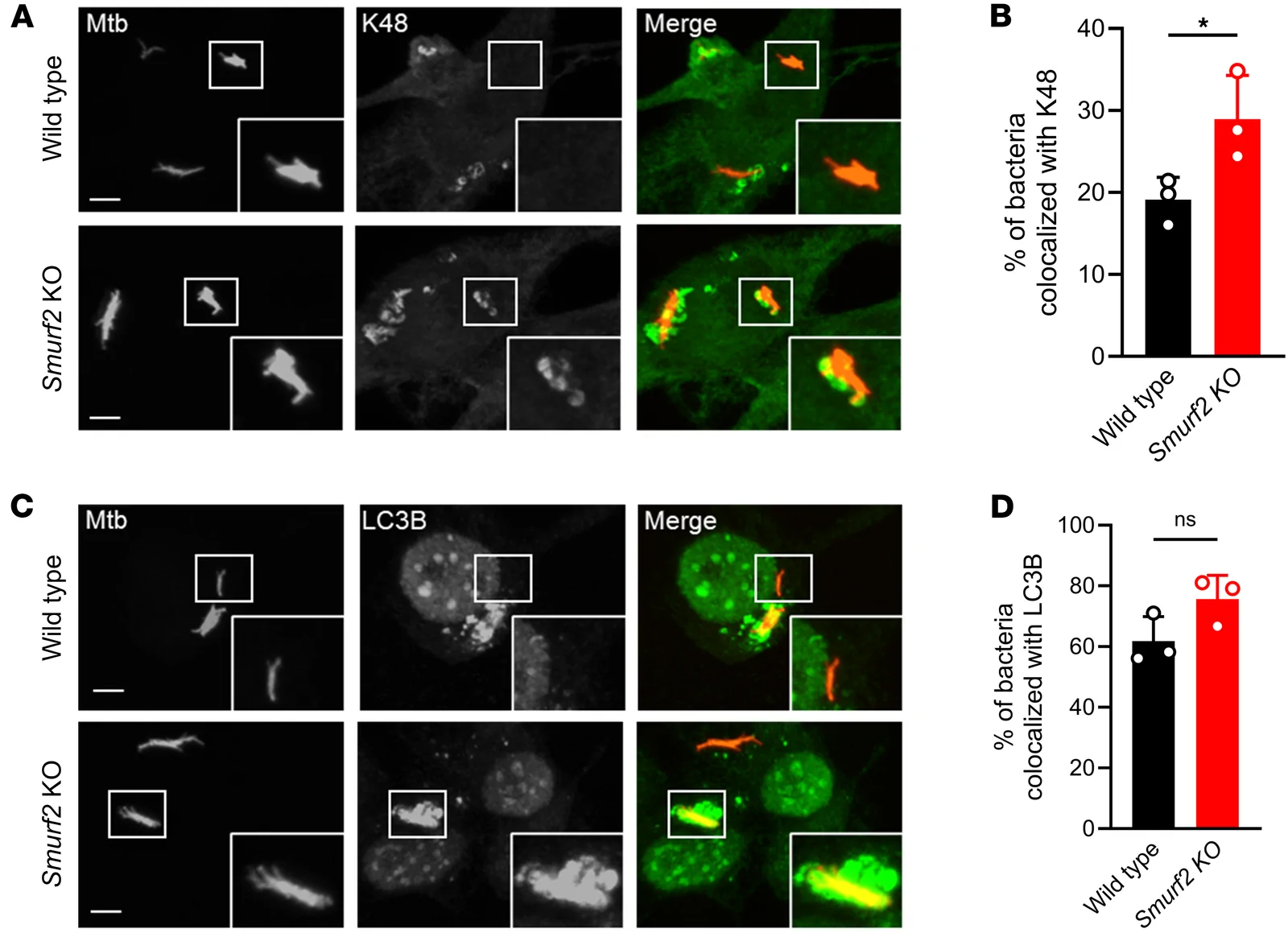
K48 and LC3B colocalize with *M*. *tuberculosis* in murine macrophages lacking *Smurf2*. (**A**) Representative immunofluorescence images of *M*. *tuberculosis*–mCherry (red) and K48 ubiquitin (green) in BV2-WT and BV2 *Smurf2*-KO cells. Images were captured 17 hours after infection using an anti-K48 antibody. (**B**) Quantification of *M*. *tuberculosis*–mCherry colocalization with K48 from **A** (mean ± SD of 3 independent experiments, performed in triplicate, *P ≤ 0.05; unpaired *t* test). (**C**) Representative immunofluorescence images of *M*. *tuberculosis*–mCherry (red) with LC3B (green) in BV2-WT and *Smurf2*-KO cells. Images were acquired 17 hours after infection using an anti-LC3B antibody. (**D**) Quantification of *M*. *tuberculosis*–mCherry (red) colocalization with LC3B (green) from **C** (mean ± SD of 3 independent experiments, performed in triplicate, ns, nonsignificant; unpaired *t* test). Scale bar: 5 μm.

**Figure 3:**
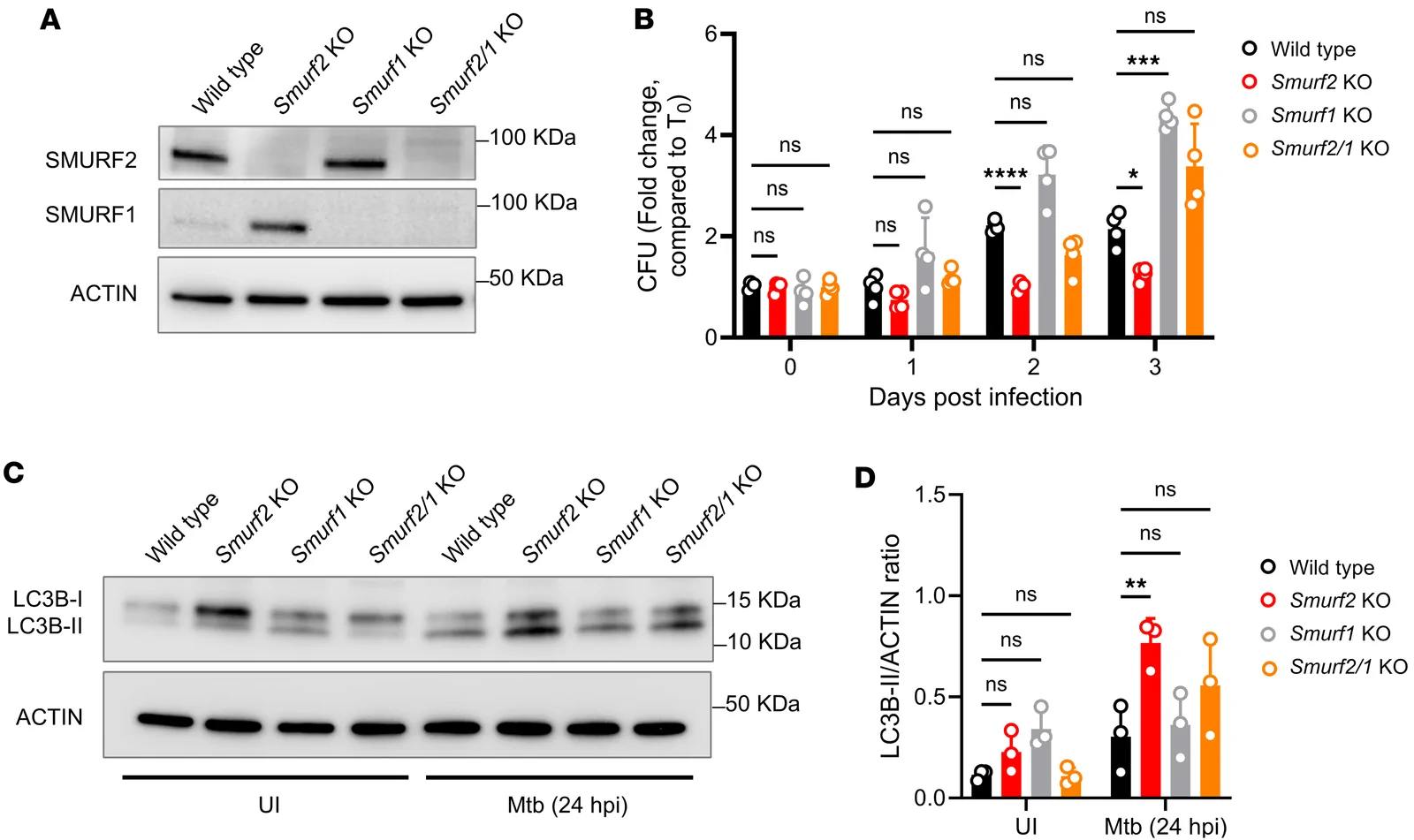
*Smurf2* KO affects *M*. *tuberculosis* replication through SMURF1 in murine macrophages. (**A**) Immunoblot analysis to detect SMURF2 and SMURF1 in BV2-WT, *Smurf2*-KO, *Smurf1*-KO, and *Smurf2/1*-KO cells under basal conditions. (**B**) CFU of *M*. *tuberculosis* in BV2-WT, *Smurf2*-KO, *Smurf1*-KO, and *Smurf2/1*-KO BV2 cells at different time points (mean ± SD of a representative experiment out of 3 independent experiments, each performed in quadruplicate, ns, nonsignificant, *P ≤ 0.05 and ***P ≤ 0.001; 2-way ANOVA, with multiple comparisons). (**C**) Immunoblot analysis of LC3B-II levels in BV2-WT, *Smurf2*-KO, *Smurf1*-KO, and *Smurf2/1*-KO cells, infected or not with *M*. *tuberculosis* (MOI 5) at 24 hours after infection. (**D**) Densitometric quantification of LC3B-II levels, as shown in **C** (mean ± SD of 3 independent experiments, ns, nonsignificant, **P ≤ 0.01; 2-way ANOVA, with multiple comparisons).

**Figure 4:**
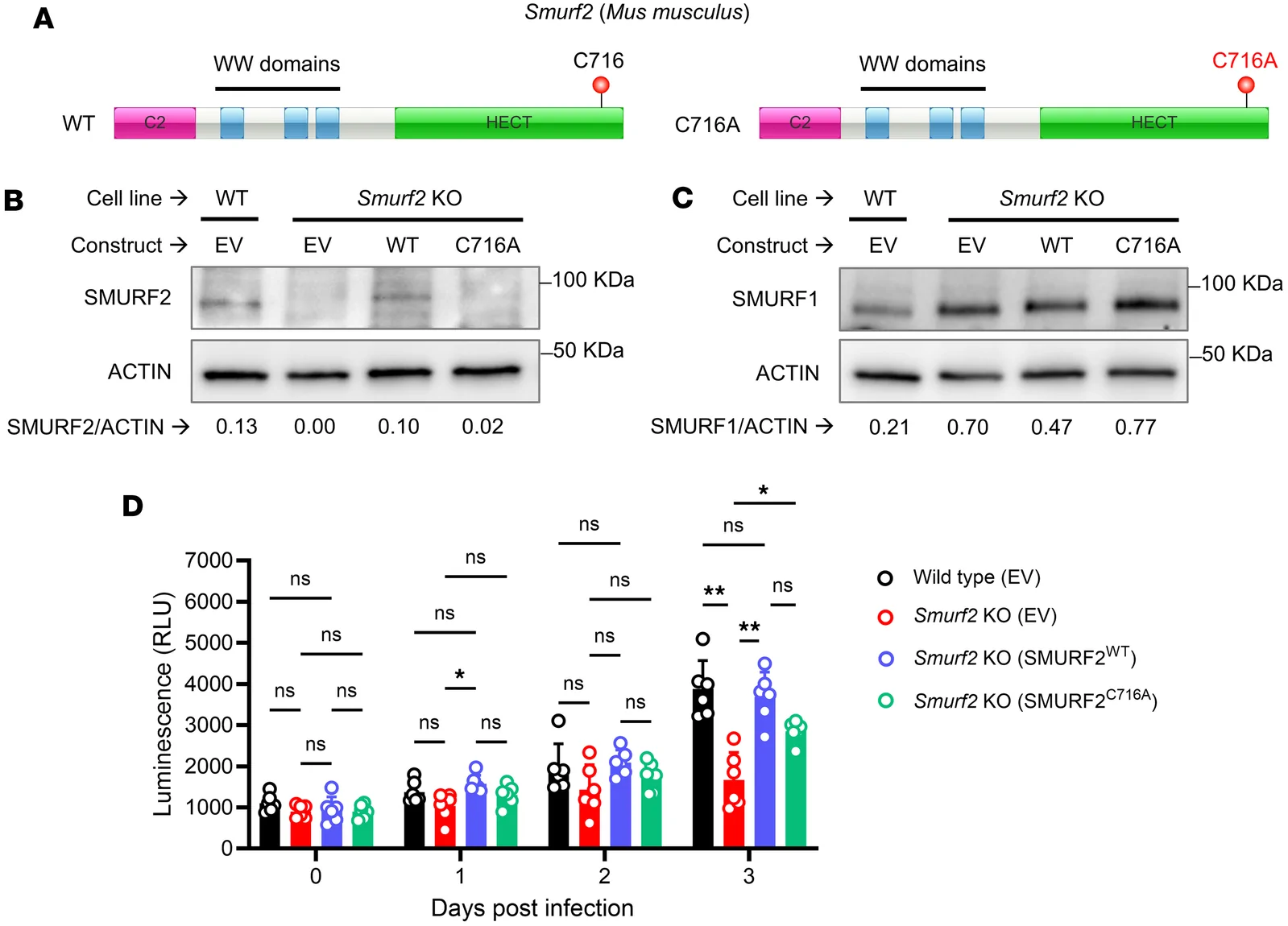
Complementation of BV2 *Smurf2*-KO cells for SMURF2 expression using a lentiviral plasmid and impact on *M*. *tuberculosis* luminescence. (**A**) Schematic of *Smurf2* cDNA constructs cloned into a lentiviral vector, for CMV-driven expression of WT (SMURF2^WT^) or mutant (SMURF2^C716A^) proteins in BV2 cells. (**B**) Immunoblot analysis of SMURF2 in BV2 cell lysates transduced with either EV or the *Smurf2* constructs shown in **A**, under basal conditions, using ACTIN as a loading control with densitometry analysis of SMURF2/ACTIN ratio. (**C**) Immunoblot analysis of SMURF1 in BV2 cell lysates transduced with EV or the *Smurf2* constructs shown in **A**, under basal conditions, using ACTIN as a loading control with densitometry analysis of SMURF1/ACTIN ratio. (**D**) Luminescence from *M*. *tuberculosis*-pLUX in BV2-WT and *Smurf2*-KO cells, transduced with EV or the *Smurf2* constructs shown in **A**. Luminescence was measured at different time points (mean ± SD of a representative experiment out of 3 independent experiments, each performed in sextuplicate. ns, nonsignificant, *P ≤ 0.05 and **P ≤ 0.01; 2-way ANOVA, with multiple comparisons).

**Figure 5:**
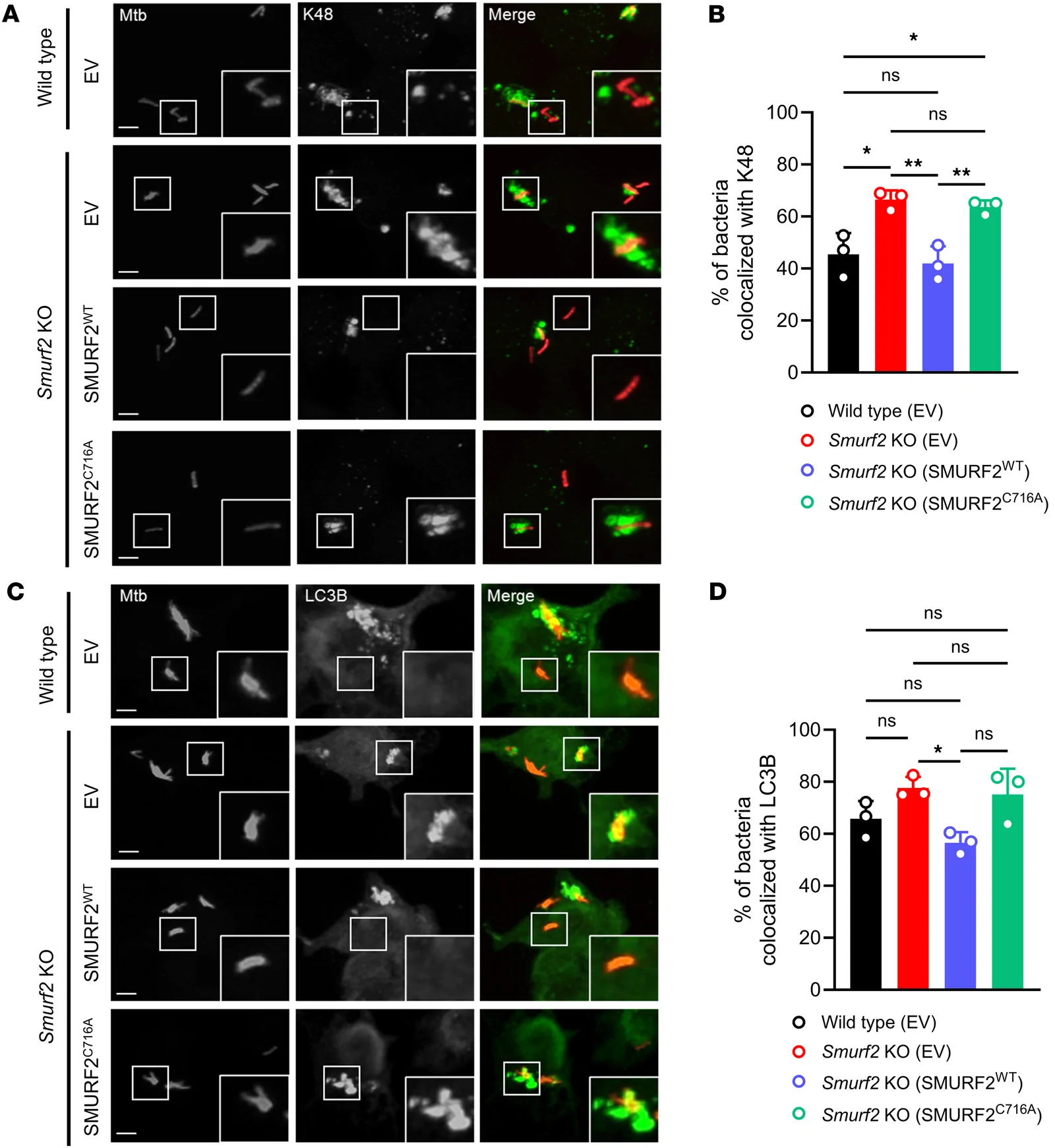
*Smurf2* complementation reduces K48 ubiquitin and LC3B colocalization with *M*. *tuberculosis* in murine macrophages. (**A**) Representative immunofluorescence images of *M*. *tuberculosis*–mCherry (red) with K48 ubiquitin (green) in BV2-WT and *Smurf2*-KO cells, transduced with either an EV or the constructs shown in Figure 4A. Cells were stained with an anti-K48 antibody 17 hours after infection. (**B**) Quantification of *M*. *tuberculosis*–mCherry colocalization with K48 as shown in **A** (mean ± SD of 3 independent experiments, performed in triplicate, ns, nonsignificant, *P ≤ 0.05 and **P ≤ 0.01; unpaired *t* test). (**C**) Representative immunofluorescence images of mCherry-expressing *M*. *tuberculosis* (red) with LC3B (green) under the same conditions as in **A**, using an anti-LC3B antibody. (**D**) Quantification of *M*. *tuberculosis*–mCherry colocalization with LC3B as shown in **C** (mean ± SD of 3 independent experiments, performed in triplicate, ns, nonsignificant, *P ≤ 0.05; unpaired *t* test). Scale bar: 5 μm.

**Figure 6:**
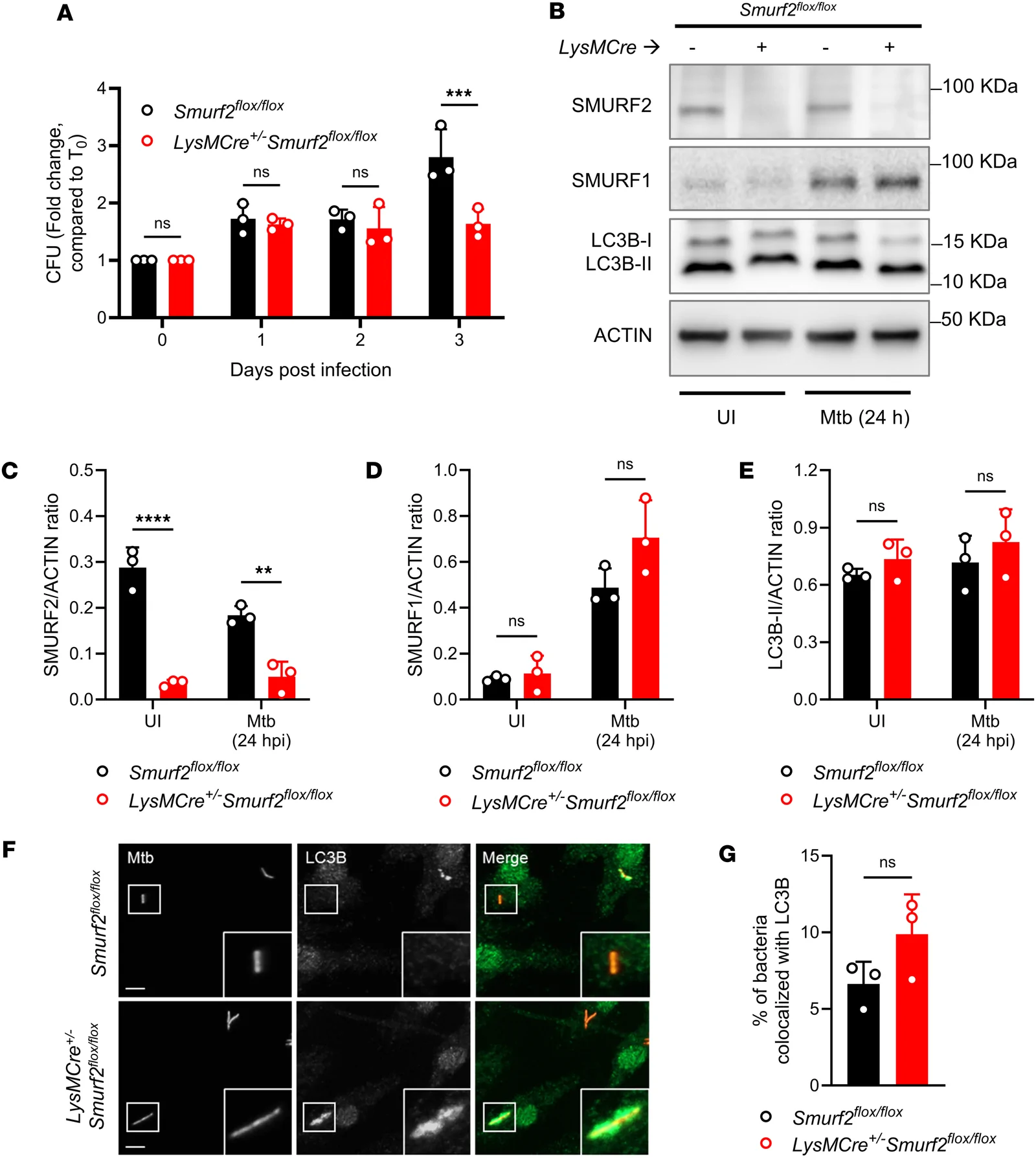
*LysMCre^+/–^Smurf2^fl/fl^* murine macrophages are more resistant to *M*. *tuberculosis* infection. (**A**) CFUs of *M*. *tuberculosis* in *Smurf2^fl/fl^* and *LysMCre^+/–^Smurf2^fl/fl^* BMDMs at different time points after infection (ns, nonsignificant, and ***P < 0.001; 2-way ANOVA). (**B**) Representative immunoblot analysis showing SMURF2, SMURF1, and LC3B lipidation levels in protein lysates from *Smurf2^fl/fl^* and *LysMCre^+/–^Smurf2^fl/fl^* BMDMs, 24 hours after infection. (**C**–**E**) Densitometric quantification of SMURF2, SMURF1, and LC3B-II levels as shown in **B**, from 3 independent experiments (mean ± SD of 3 independent experiments, ns, nonsignificant, **P < 0.01 and ****P < 0.0001; 2-way ANOVA). (**F**) Representative immunofluorescence images of *M*. *tuberculosis*–mCherry (red) with LC3B (green) in *Smurf2^fl/fl^* and *LysMCre^+/–^Smurf2^fl/fl^* BMDMs, using anti-LC3B antibody 17 hours after infection. (**G**) Quantification of *M*. *tuberculosis*–mCherry (red) colocalization with LC3B (green) as shown in **F** (mean ± SD of 3 independent experiments, performed in triplicate, ns, nonsignificant; unpaired *t* test). Scale bar: 5 μm.

**Figure 7:**
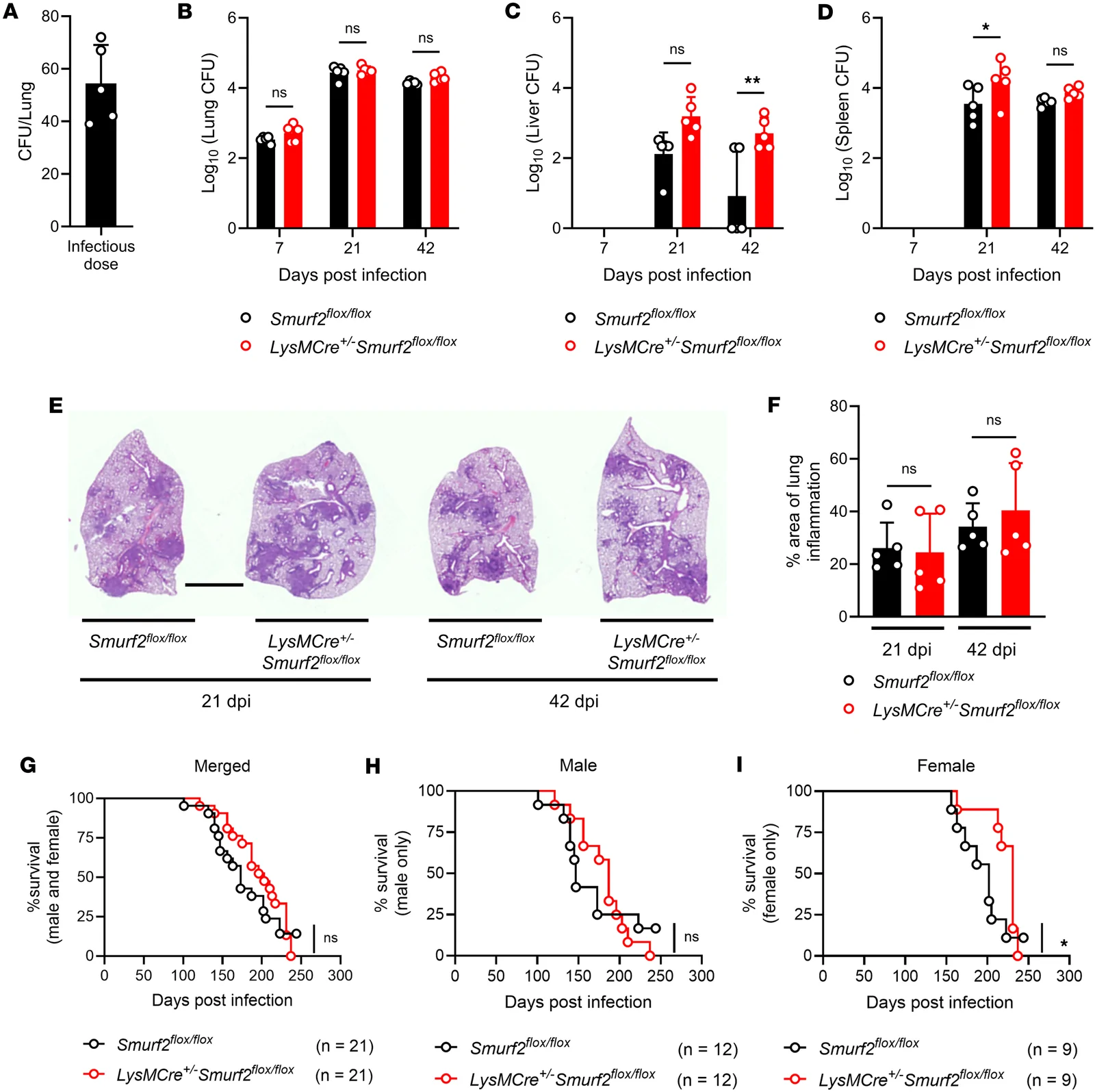
*Smurf2* deficiency does not significantly affect *M*. *tuberculosis* infection in vivo. (**A**) *Smurf2^fl/fl^* and *LysMCre^+/–^Smurf2^fl/fl^* mice were aerosol-infected with approximately 50 CFU Erdman *M*. *tuberculosis*. Mice were euthanized at different time points, and CFUs were enumerated in the (**B**) lungs, (**C**) liver, and (**D**) spleen at 7, 21, and 42 days after infection (dpi) (*n* = 5 mice/group, mean ± SD of an experiment performed in quadruplicate, ns, nonsignificant; 2-way ANOVA). (**E**) Representative H&E staining of lung sections from *M*. *tuberculosis*–infected mice at 21 and 42 dpi. Scale bar: 2.5 mm. (**F**) Quantification of inflammatory areas in lung sections at 21 and 42 dpi, as shown in **E**. Data represent mean ± SD (*n* = 5 mice/group, ns, nonsignificant; Mann-Whitney *U* test). (**G**–**I**) Kaplan-Meier survival curves of *Smurf2^fl/fl^* and *LysMCre^+/–^Smurf2^fl/fl^ M*. *tuberculosis*–infected mice (*n* = 21 animals/genotype), shown for (**G**) male and female mice, (**H**) male only, and (**I**) female only (ns, nonsignificant; *P ≤ 0.05; log-rank test).

**Figure 8:**
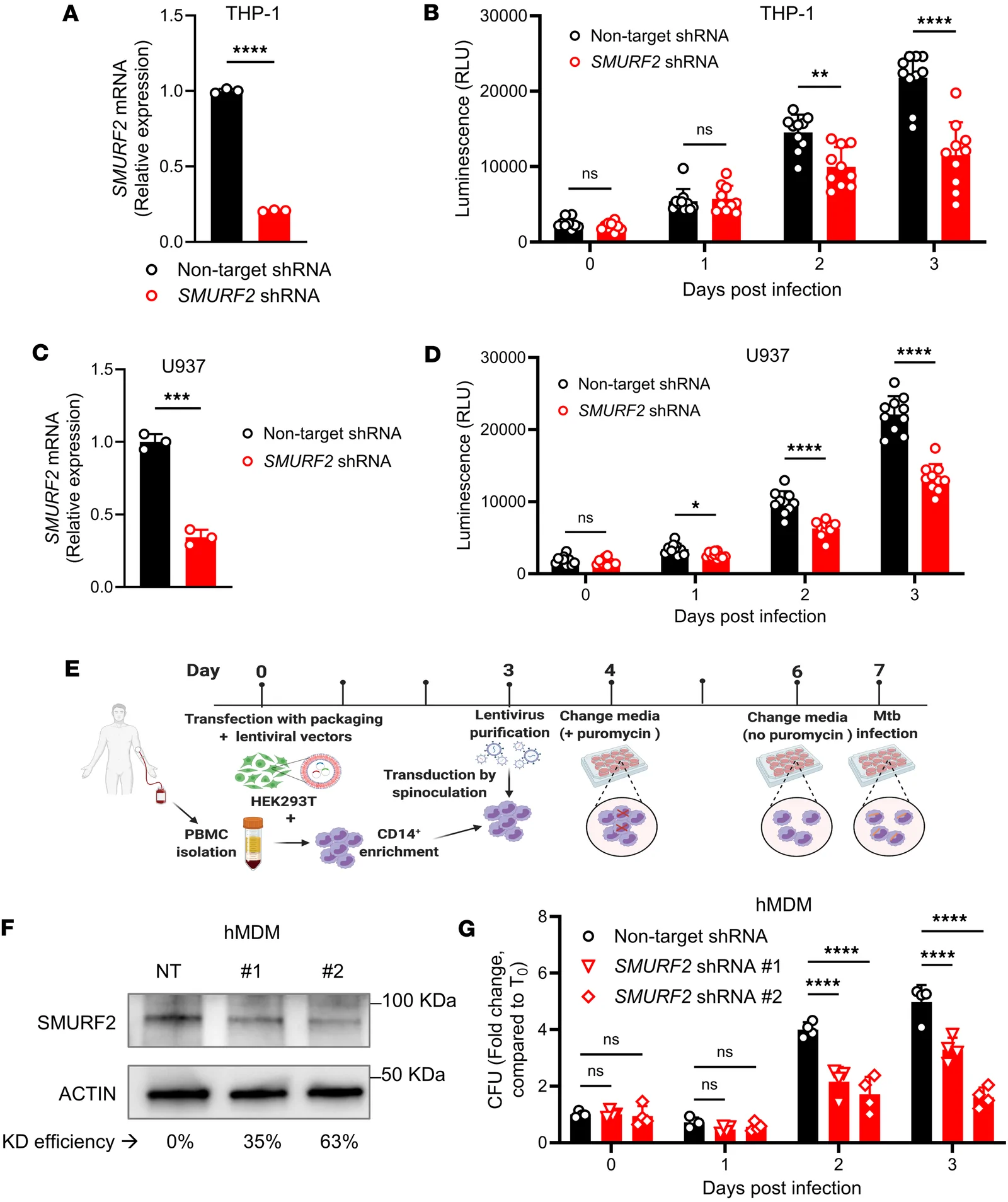
*SMURF2* knockdown reduces *M*. *tuberculosis* intracellular growth in human macrophages. (**A**) Relative mRNA expression of *SMURF2* in THP-1 cells, quantified by reverse transcription-qPCR (RT-qPCR) and normalized to *GAPDH* gene. Data are representative of 3 independent experiments performed in triplicate (****P ≤ 0.0001; unpaired *t* test). (**B**) Luminescence from *M*. *tuberculosis*–pLUX in THP-1 cells knocked down for the *SMURF2* gene. Luminescence was measured at different time points (mean ± SD of a representative experiment performed with 10 technical replicates. ns, nonsignificant, **P ≤ 0.01 and ****P ≤ 0.0001; 2-way ANOVA). (**C**) Relative mRNA expression of *SMURF2* in U937 cells, quantified as in **A**. Data are representative of 3 independent experiments performed in triplicate (***P ≤ 0.001; unpaired *t* test). (**D**) Luminescence from *M*. *tuberculosis*–pLUX in U937 cells knocked down for the *SMURF2* gene. Luminescence was measured as in **B** (mean ± SD of a representative experiment performed with 10 technical replicates. ns, nonsignificant, *P ≤ 0.05 and ****P ≤ 0.0001; 2-way ANOVA). (**E**) Schematic of the isolation and transduction of CD-14^+^–enriched hMDMs (see Methods for details). (**F**) Representative immunoblot of SMURF2 in hMDMs from a single donor transduced with nontargeting shRNA (NT) or 2 different *SMURF2* shRNAs (1 or 2). SMURF2 knockdown efficiencies are indicated below ACTIN bands. (**G**) Intracellular *M*. *tuberculosis* CFU in hMDMs transduced as in **E** with SMURF2 shRNA (1 or 2) at different time points after infection (mean ± SD of a representative experiment out of 2 independent experiments, each performed in quadruplicate. ns, nonsignificant, **P ≤ 0.01 and ***P ≤ 0.001; 2-way ANOVA).

**Figure 9:**
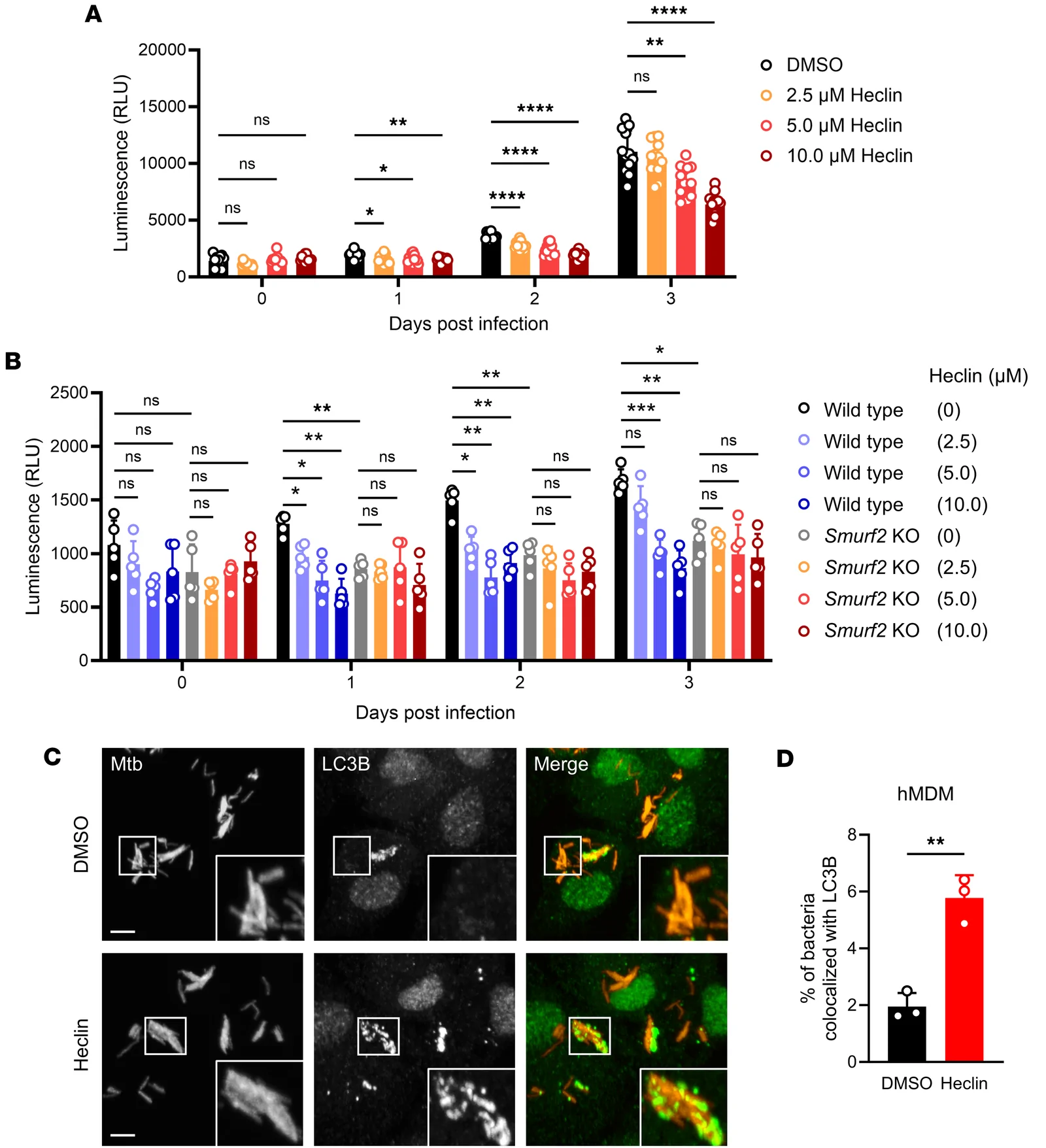
Inhibition of SMURF2 by Heclin reduces *M*. *tuberculosis* intracellular growth in human macrophages. (**A**) Luminescence from *M*. *tuberculosis*–pLUX in hMDMs exposed to either 0.1% DMSO (vehicle control) or increasing concentrations of Heclin, as indicated (see Methods for details). Luminescence was measured at different time points (mean ± SD of a representative experiment out of 2 independent experiments, each performed in 12 replicates, ns, nonsignificant, *P ≤ 0.05, **P ≤ 0.01, and ****P ≤ 0.0001; 2-way ANOVA). (**B**) Luminescence from *M*. *tuberculosis*–pLUX in BV2-WT and *Smurf2*-KO cells exposed to either 0.1% DMSO (vehicle control) or increasing concentrations of Heclin. Luminescence was measured at different time points (mean ± SD of a representative experiment out of 2 independent experiments, each performed in quintuplicate. ns, nonsignificant, *P ≤ 0.05, **P ≤ 0.01, and ***P ≤ 0.001; 2-way ANOVA, with multiple comparisons). (**C**) Representative immunofluorescence images of *M*. *tuberculosis*–mCherry (red) and LC3B (green) in human macrophages exposed to either 0.1% DMSO (vehicle control) or 10 μM Heclin. (**D**) Quantification of *M*. *tuberculosis*–mCherry colocalization with LC3B shown in **C** (mean ± SD of 3 independent experiments performed in triplicate, **P ≤ 0.01; unpaired *t* test). Scale bar: 5 μm.

## Version 1 09/08/2026

In-Press Preview

## Version 2 10/22/2026

Electronic publication
