# Supplementary material for: SMURF2 inhibits autophagic control of Mycobacterium tuberculosis in macrophages
Source: JCI Insight. 2026 Sep 8;11(20):e199853. doi: 10.1172/jci.insight.199853 (PMC13726567; doi:10.1172/jci.insight.199853)
Supplement: Supplementary file 1 [file jciinsight-11-199853-s126.pdf]

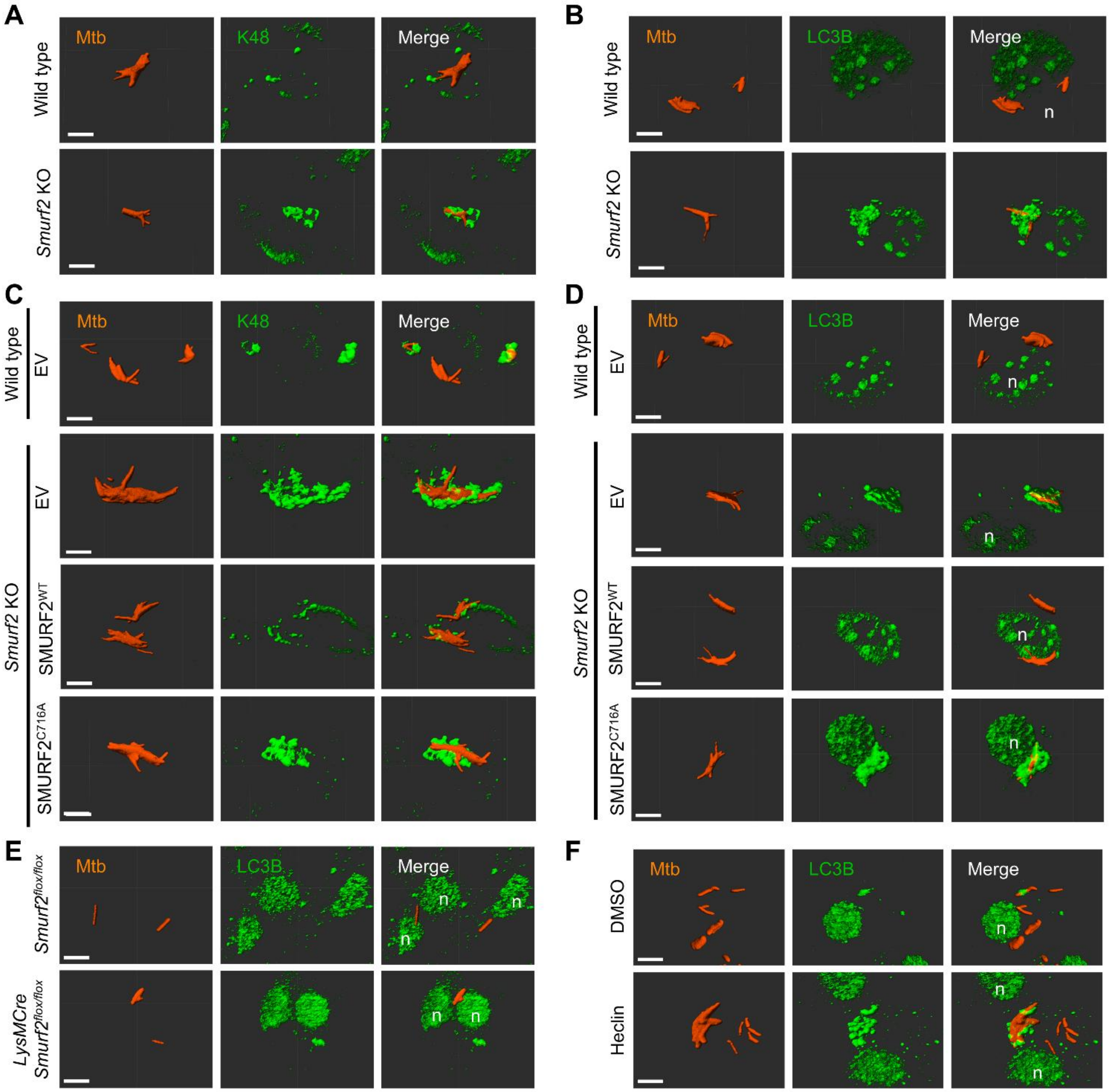

Supplemental Figure 1. 3D structural analysis of Mtb colocalization with K48-linked ubiquitin and LC3B within macrophages. Representative 3D renderings of *M. tuberculosis*-mCherry (red) colocalizing with K48-linked ubiquitin or LC3B (green). Nuclei in LC3B-stained cells are indicated by the letter 'n'. **(A-B)** Analysis in BV2 WT and BV2 *Smurf2*-KO cells, related to **Figure 2A** and **2C**. **(C-D)** Analysis in BV2 WT and BV2 *Smurf2*-KO cells transduced with empty vector (EV) or the constructs shown in **Figure 4**, related to **Figure 5A** and **5C**. **(E)** Analysis in *Smurf2*$^{flox/flox}$ and *LysMCre*$^{+/-}$*Smurf2*$^{flox/flox}$ BMDMs, relating to **Figure 6F**. **(F)** Analysis in hMDMs, relating to **Figure 9C**. Scale bar: 5 µm.

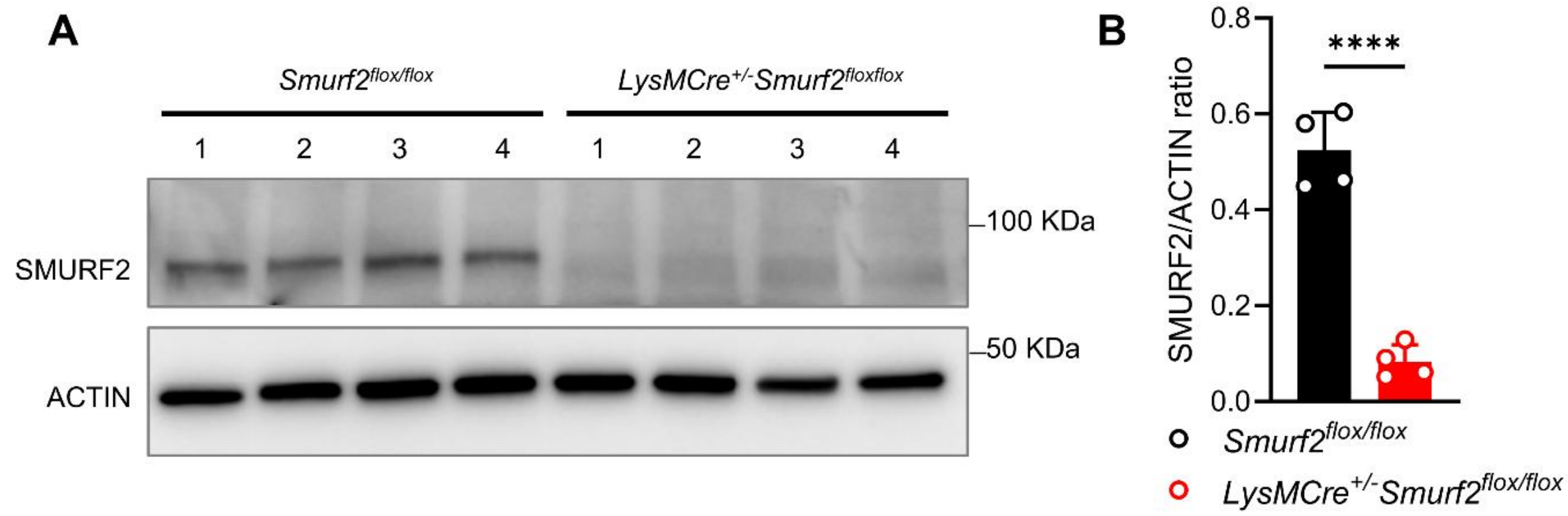

Supplemental Figure 2: SMURF2 expression in *Smurf2$^{flox/flox}$* and *LysMCre$^{+/-}$Smurf2$^{flox/flox}$* in murine bone-marrow-derived macrophages (BMDMs). (A) Representative immunoblot analysis of SMURF2 in protein lysates from *Smurf2$^{flox/flox}$* and *LysMCre$^{+/-}$Smurf2$^{flox/flox}$* BMDMs from four mice per genotype, under basal conditions. **(B)** Densitometric quantification of SMURF2 levels shown in **(A)** (n = 4 mice/group, ****p < 0.0001; unpaired t test).

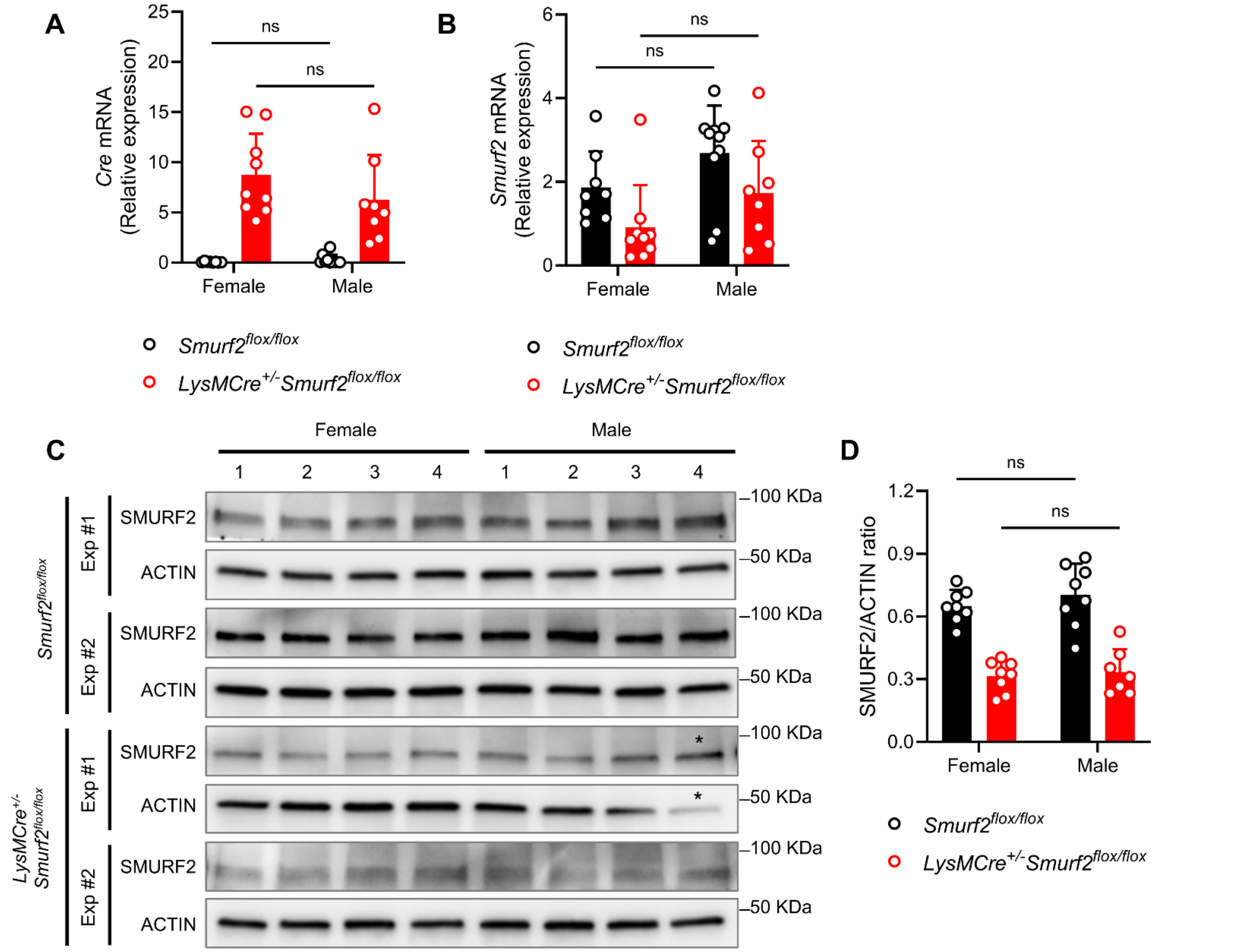

Supplemental Figure 3. Increased female survival *in vivo* is not driven by sex-specific differences in SMURF2 expression in BMDMs. (A-B) Relative mRNA expression of *Cre* **(A)** and *Smurf2* **(B)** in BMDMs derived from female and male *Smurf2$^{flox/flox}$* and *LysMCre$^{+/-}$Smurf2$^{flox/flox}$* mice. Levels were quantified by RT-qPCR and normalized to 18S rRNA. Data represent 8 mice per sex per genotype (ns, nonsignificant; 2-way ANOVA). **(C)** Immunoblot analysis of SMURF2 in protein lysates from *Smurf2$^{flox/flox}$* and *LysMCre$^{+/-}$Smurf2$^{flox/flox}$* BMDMs under basal conditions. Two independent experiments are shown, each including 4 mice per sex per genotype. **(D)** Densitometric quantification of SMURF2 levels shown in **(C)**. Data represent 8 mice per sex per genotype pooled from the 2 independent experiments shown in **(C)** (ns, nonsignificant; 2-way ANOVA). Asterisks in lane 8 indicate bands excluded from quantification because of a technical artifact during protein transfer.

A

B

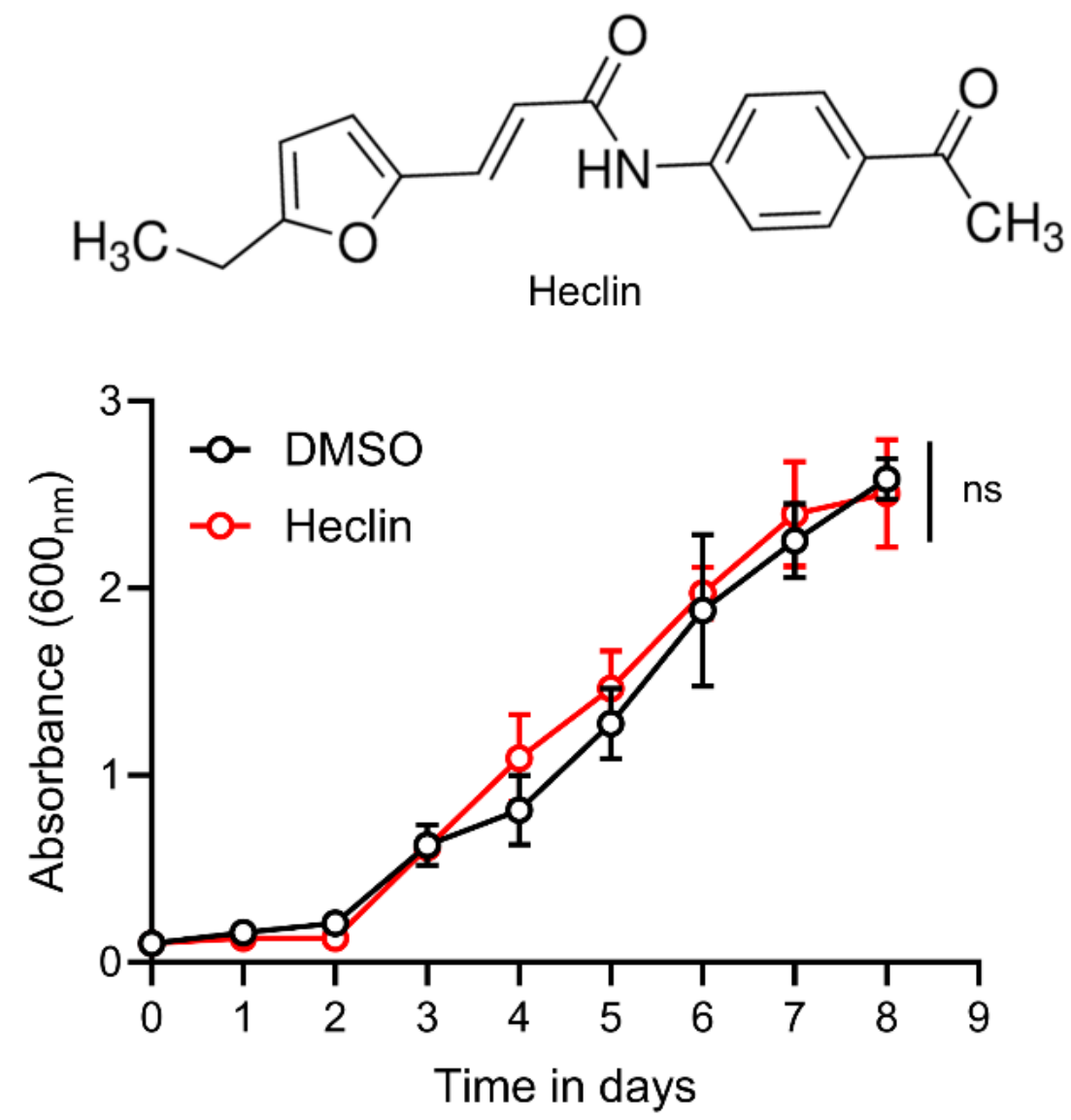

Supplemental Figure 4: Mtb growth in the presence of DMSO or Heclin. (A) Chemical structure of the HECT E3 ubiquitin ligase inhibitor, Heclin (N-(4-Acetylphenyl)-3-(5-ethyl-2-furanyl)-2-propenamide). **(B)** Growth curves of Mtb Erdman in 7H9 medium, supplemented with 10% OADC, 0.5% glycerol, and 0.05% Tween-80, exposed to either 0.1% DMSO (vehicle control) or 10 µM Heclin. OD_{600} was measured at different time points. Data represent mean ± SD from three independent cultures per condition (ns, nonsignificant; 2-way ANOVA).
