# Supplementary material for: SMURF2 inhibits autophagic control of Mycobacterium tuberculosis in macrophages
Source: JCI Insight. 2026 Sep 8;11(20):e199853. doi: 10.1172/jci.insight.199853 (PMC13726567; doi:10.1172/jci.insight.199853)

Full unedited gels for Figure 1A

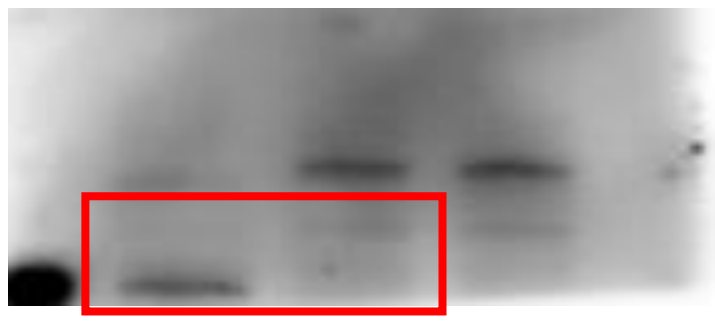

SMURF2
(Replicate 1)

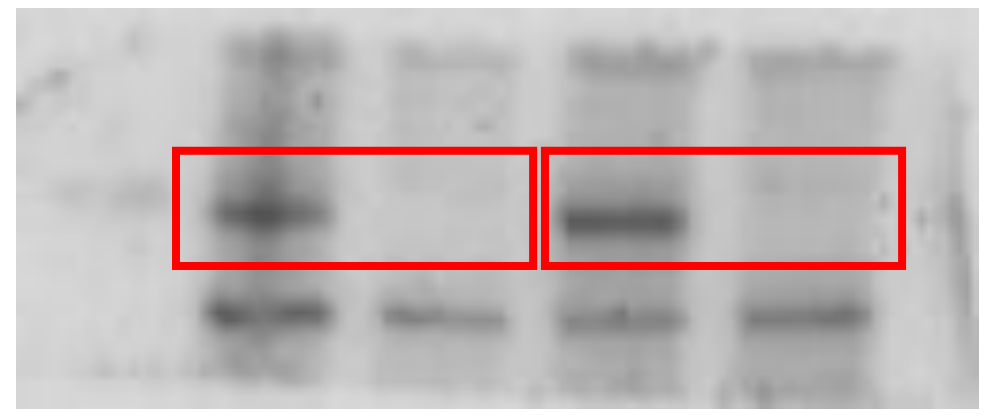

SMURF2
(Replicate 2)

SMURF2
(Replicate 3)

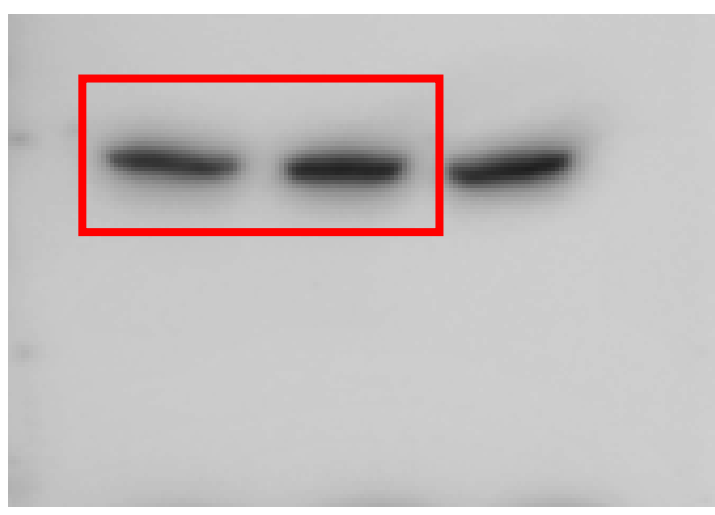

ACTIN
(Replicate 1)

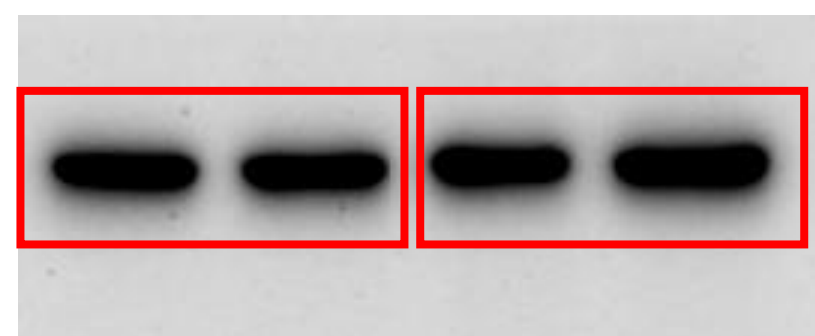

ACTIN
(Replicate 2)

ACTIN
(Replicate 3)

SMURF1
(Replicate 1)

ACTIN
(Replicate 1)

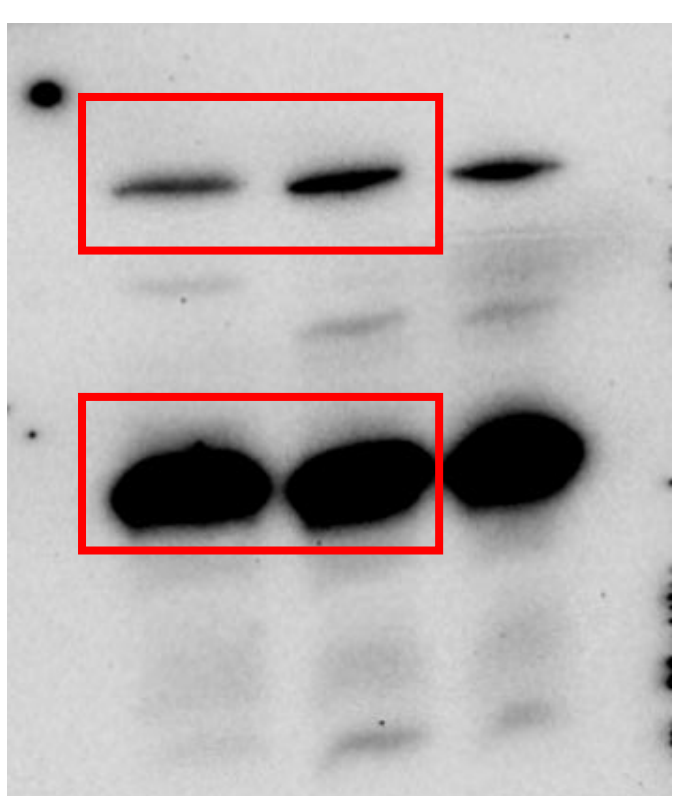

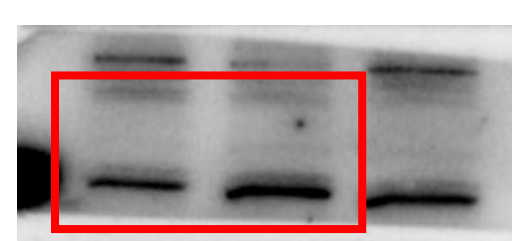

SMURF1
(Replicate 2)

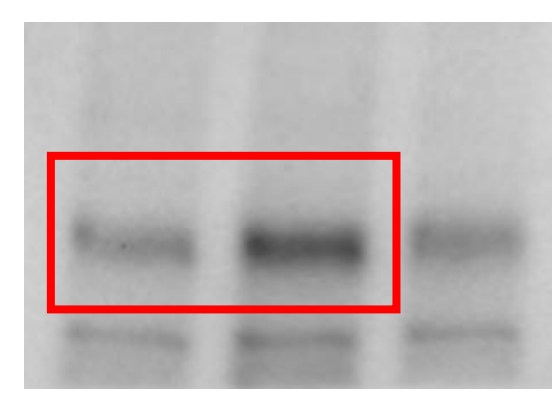

SMURF1
(Replicate 3)

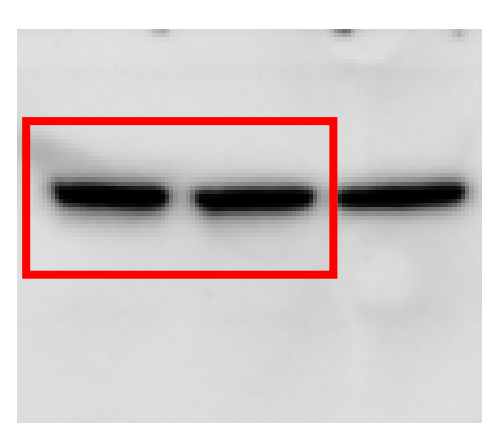

ACTIN
(Replicate 2)

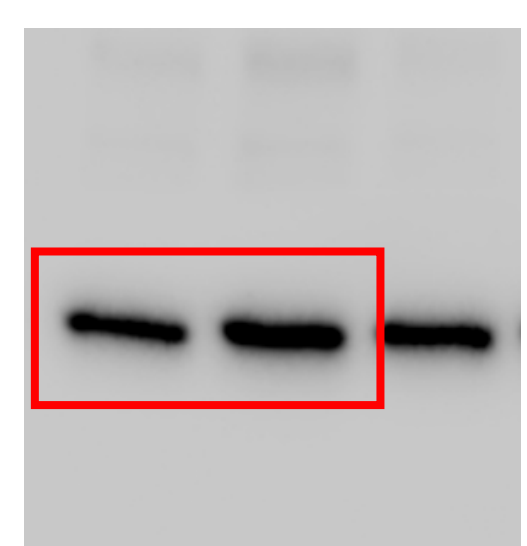

ACTIN
(Replicate 3)

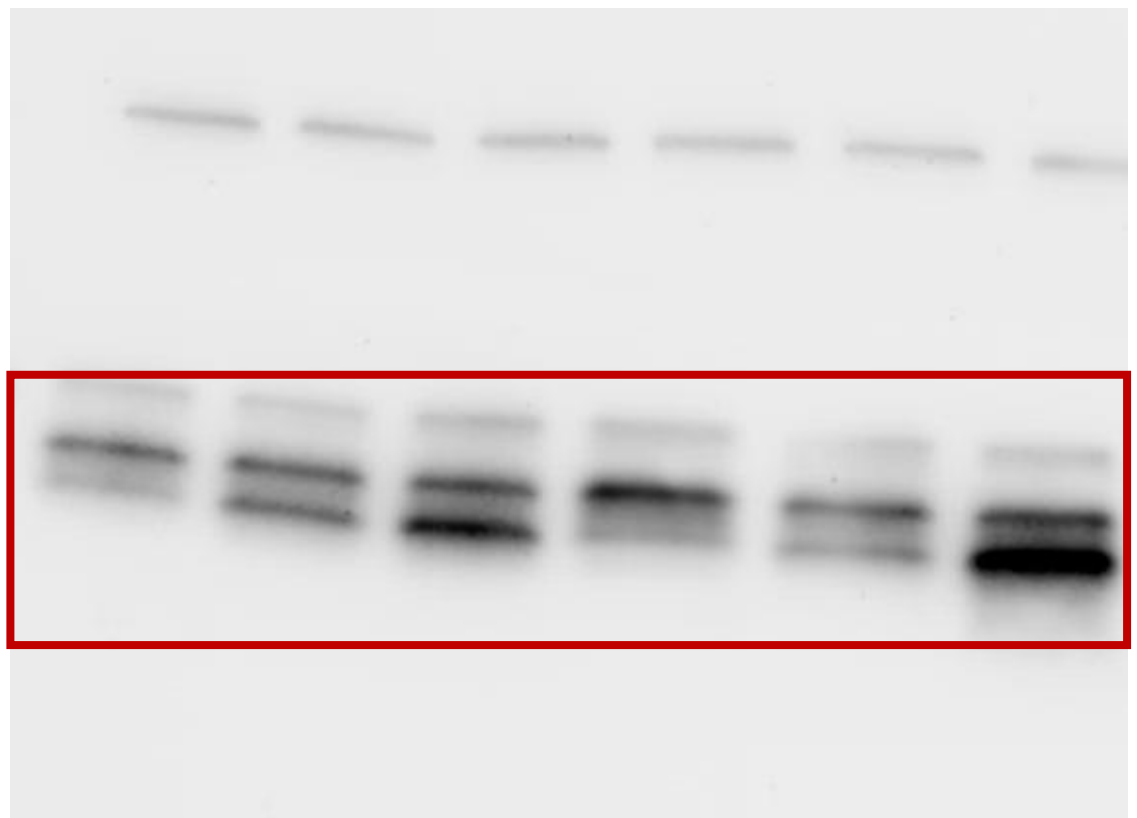

LC3B-I (top)
LC3B-II (bottom)
(Replicate 1)

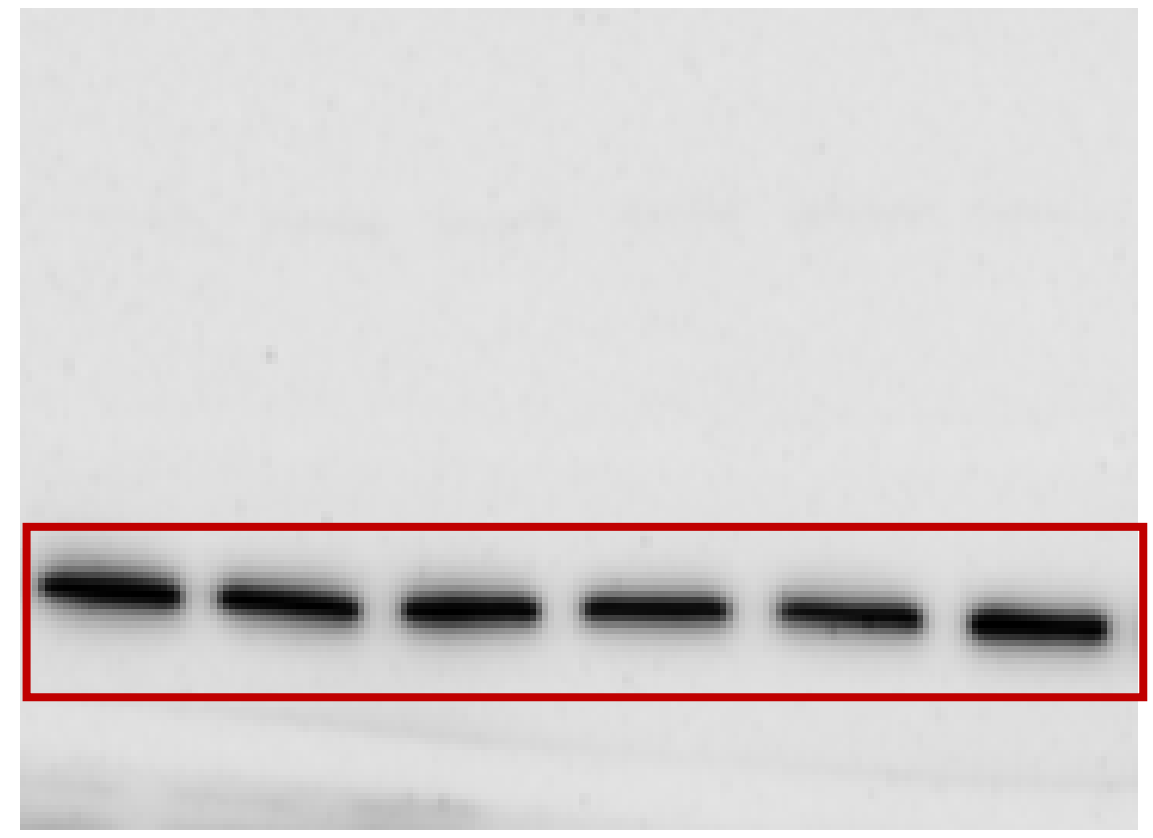

ACTIN
(Replicate 1)

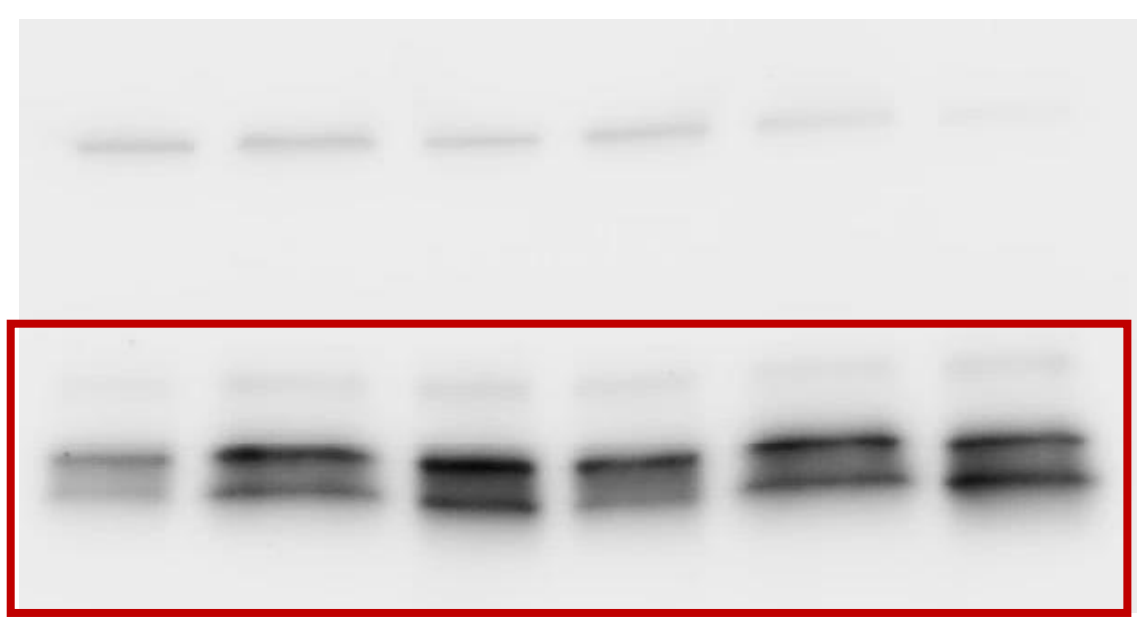

LC3B-I (top)
LC3B-II (bottom)
(Replicate 2)

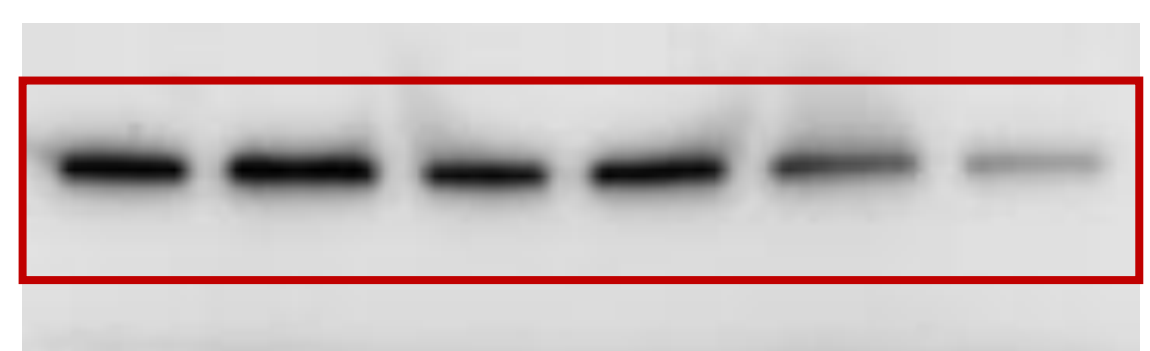

ACTIN
(Replicate 2)

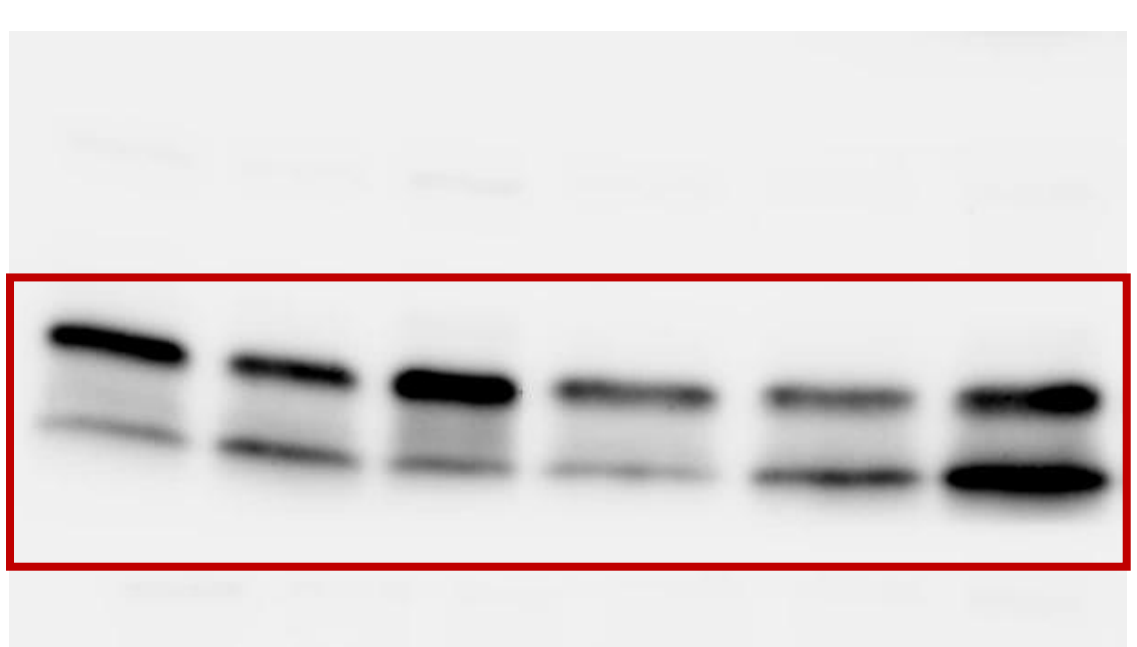

LC3B-I (top)
LC3B-II (bottom)
(Replicate 3)

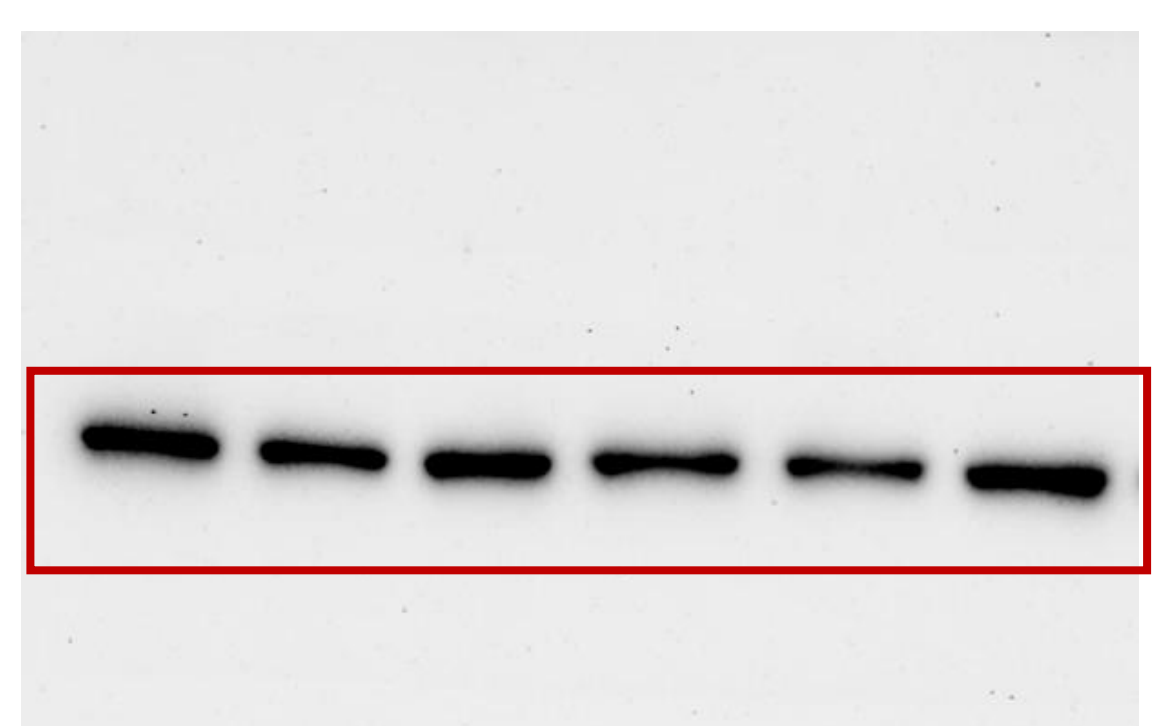

ACTIN
(Replicate 3)

Full unedited gels for Figure 3A

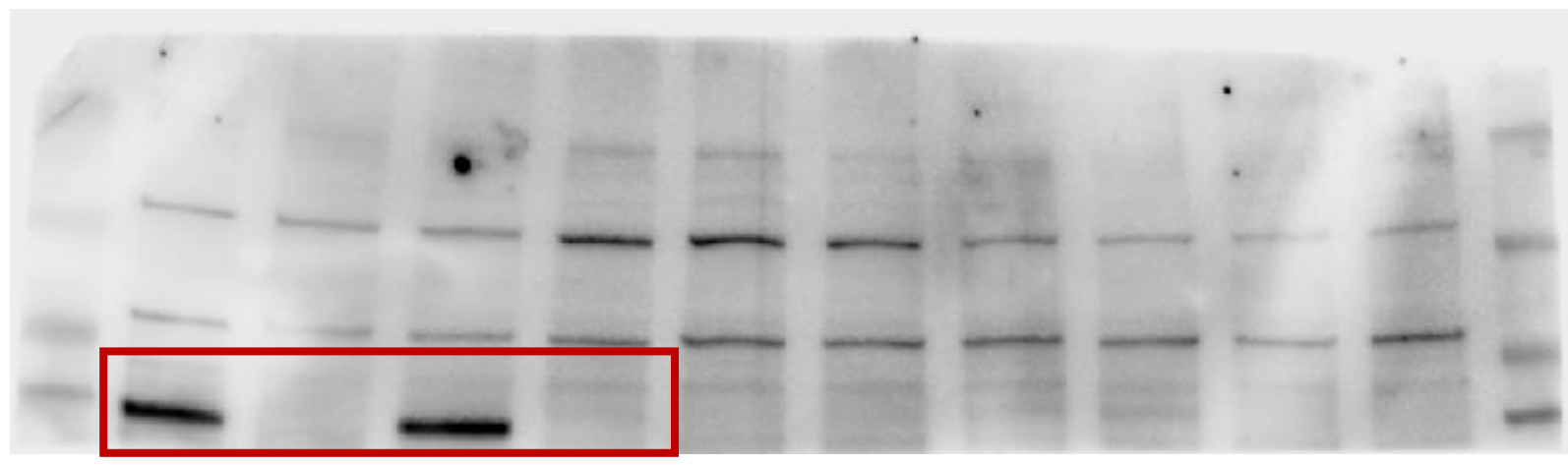

SMURF2

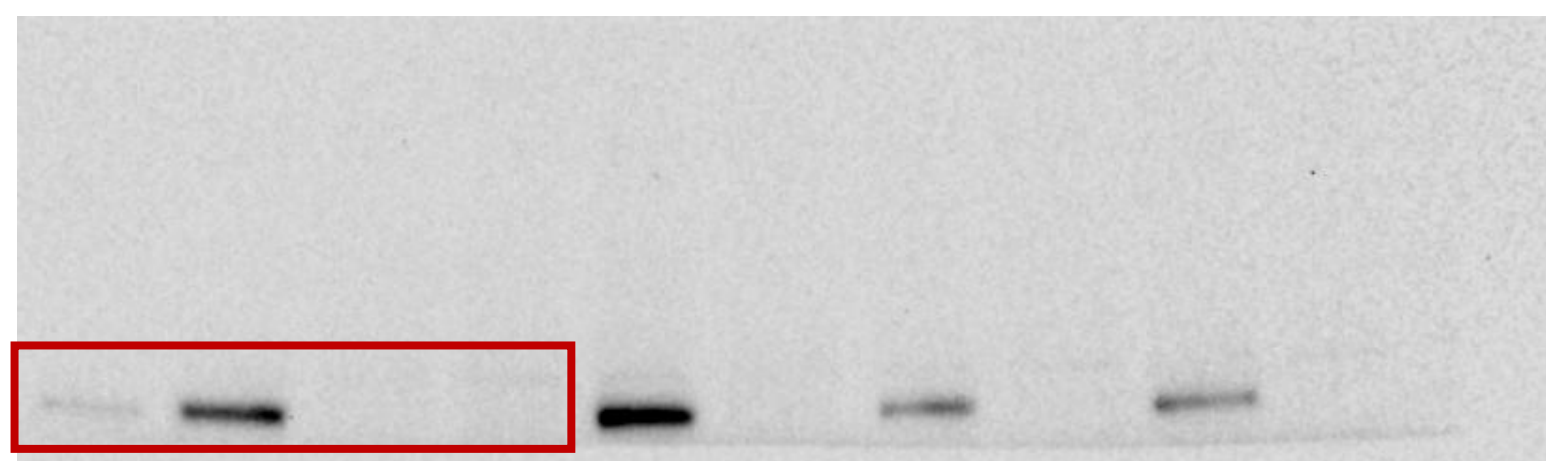

SMURF1

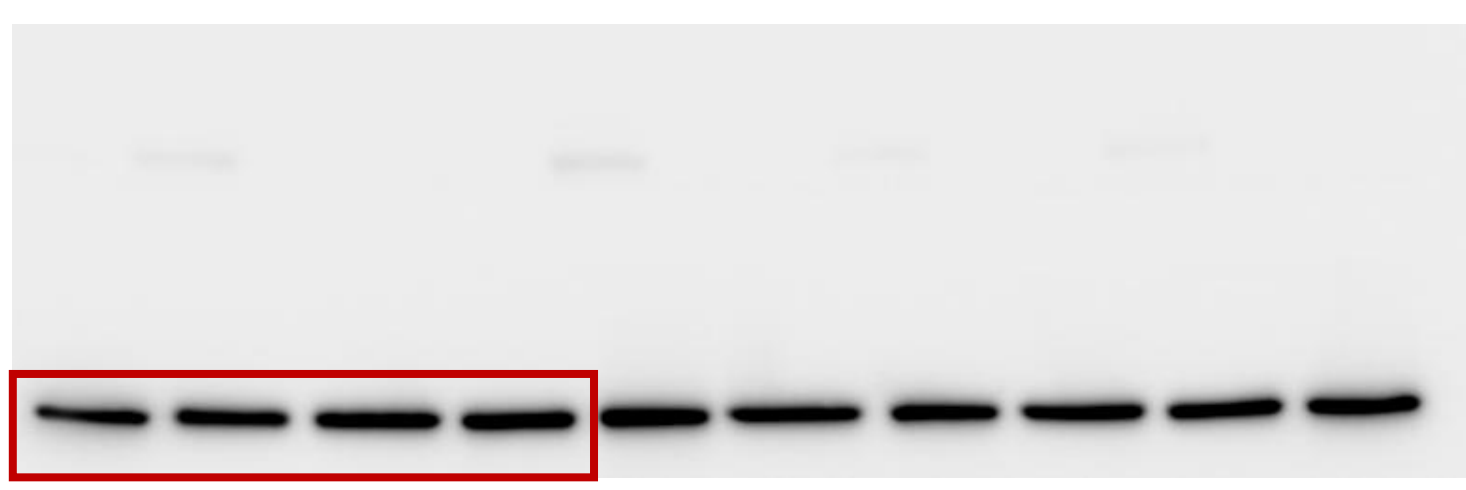

ACTIN

Full unedited gels for Figure 3C

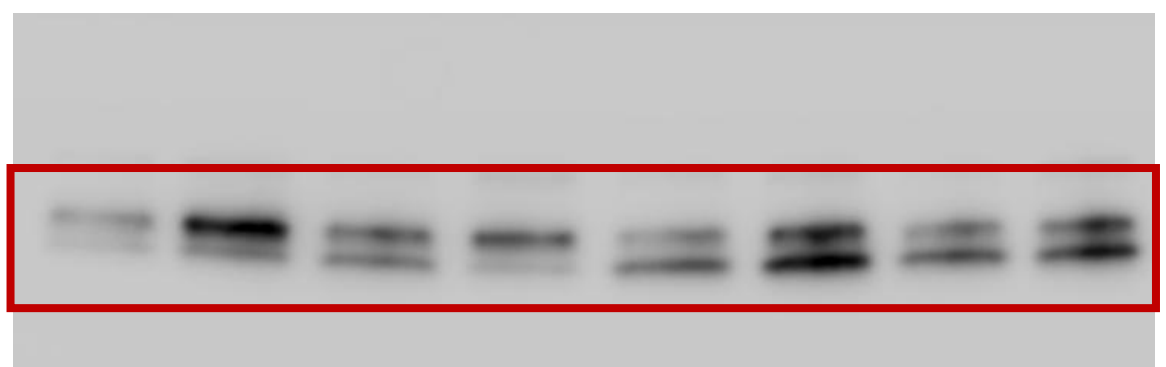

LC3B-I (top)
LC3B-II (bottom)
(Replicate 1)

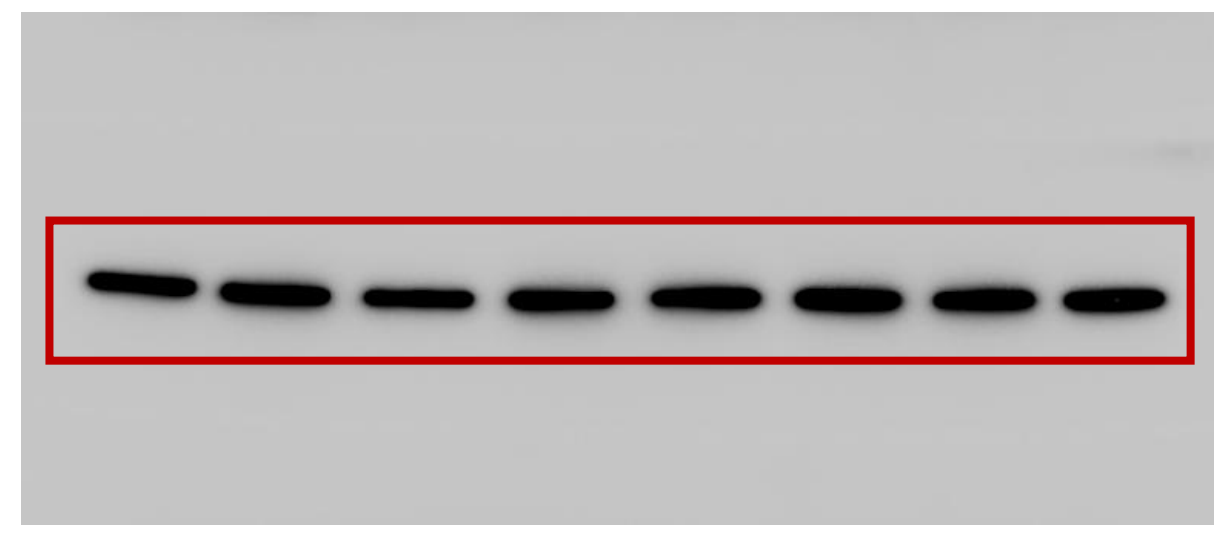

ACTIN
(Replicate 1)

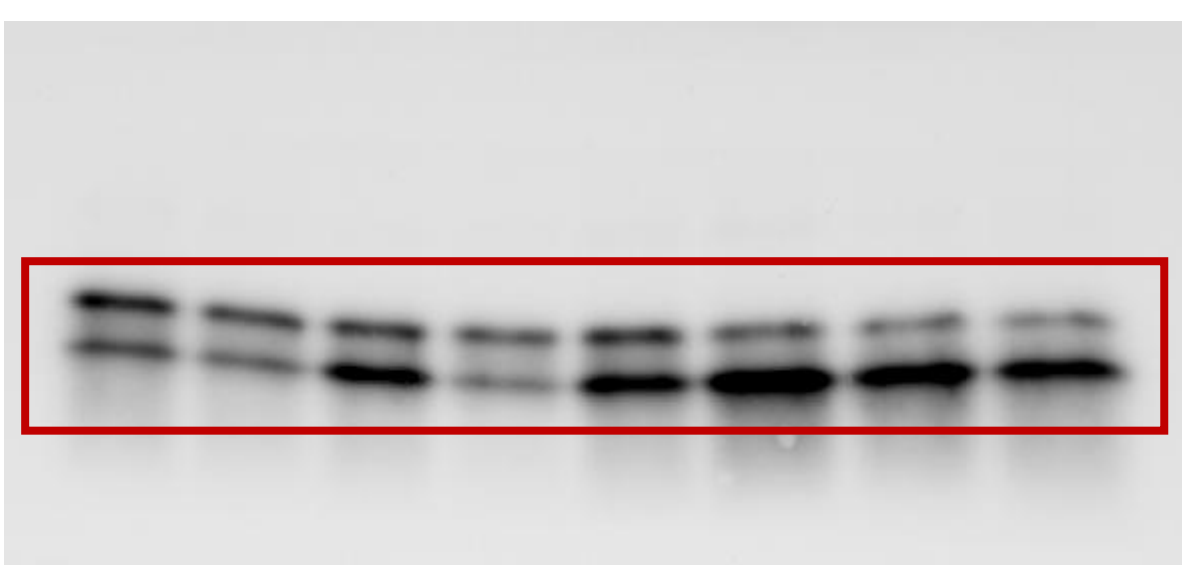

LC3B-I (top)
LC3B-II (bottom)
(Replicate 2)

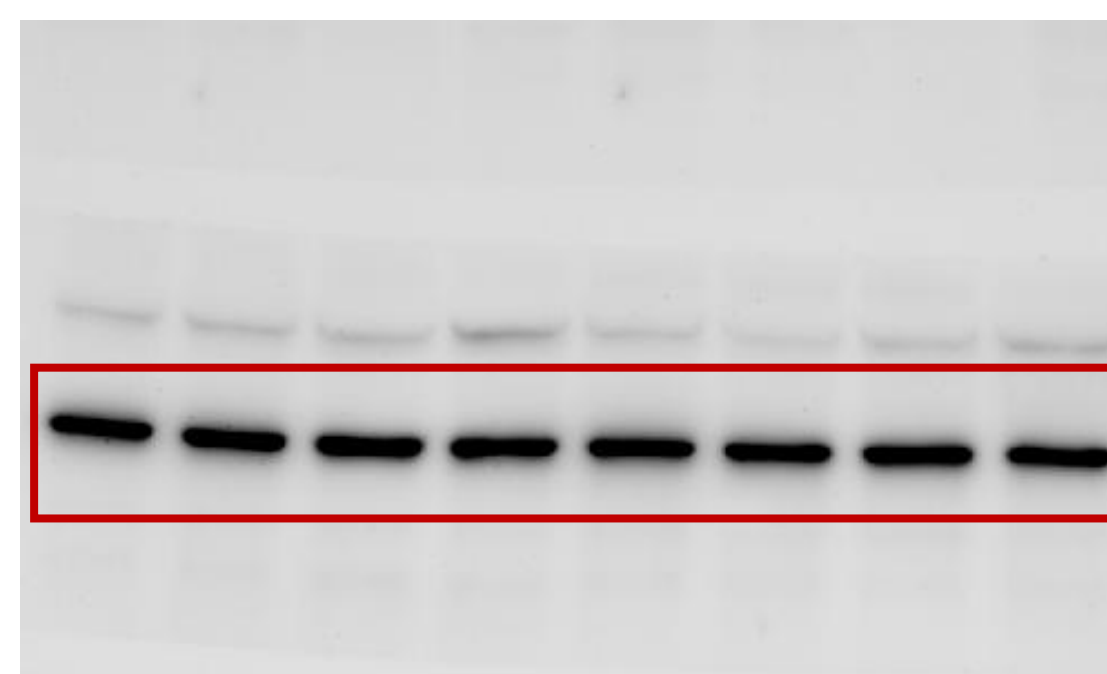

ACTIN
(Replicate 2)

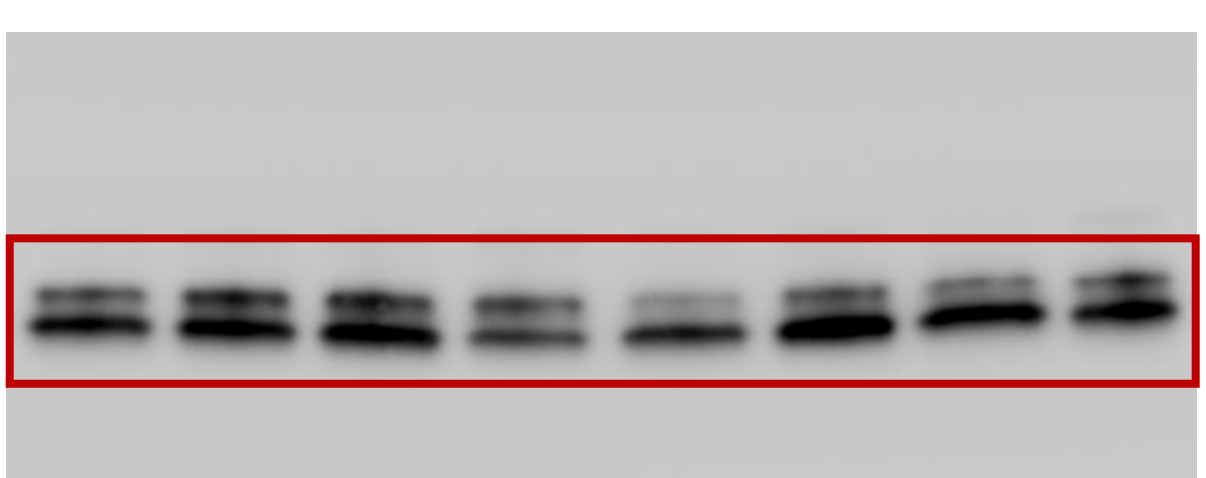

LC3B-I (top)
LC3B-II (bottom)
(Replicate 3)

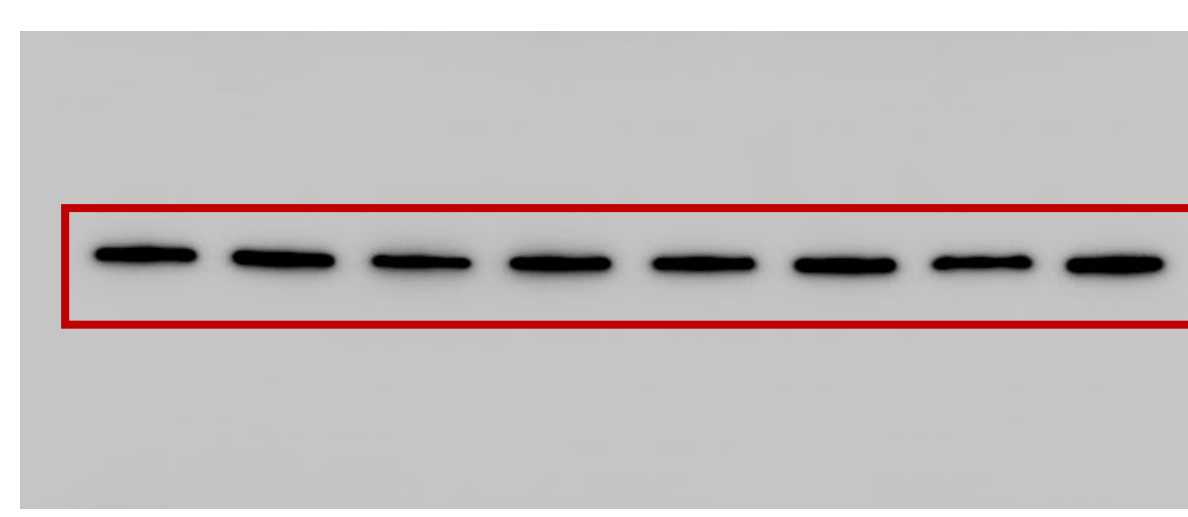

ACTIN
(Replicate 3)

Full unedited gels for Figure 4B-C

4B

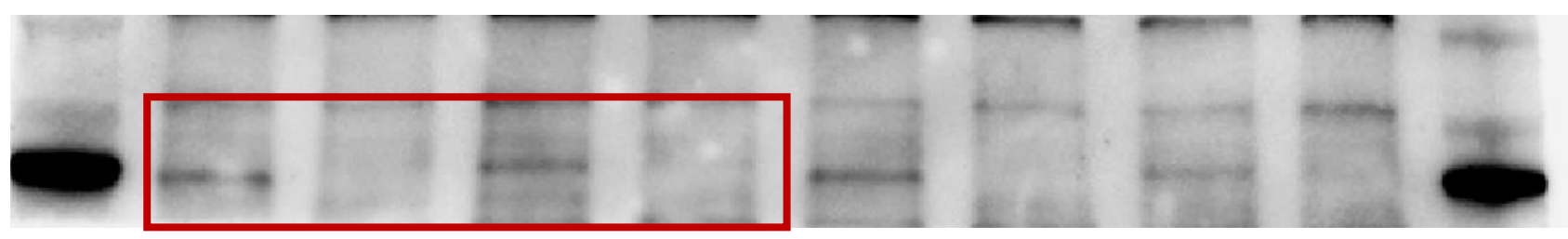

SMURF2

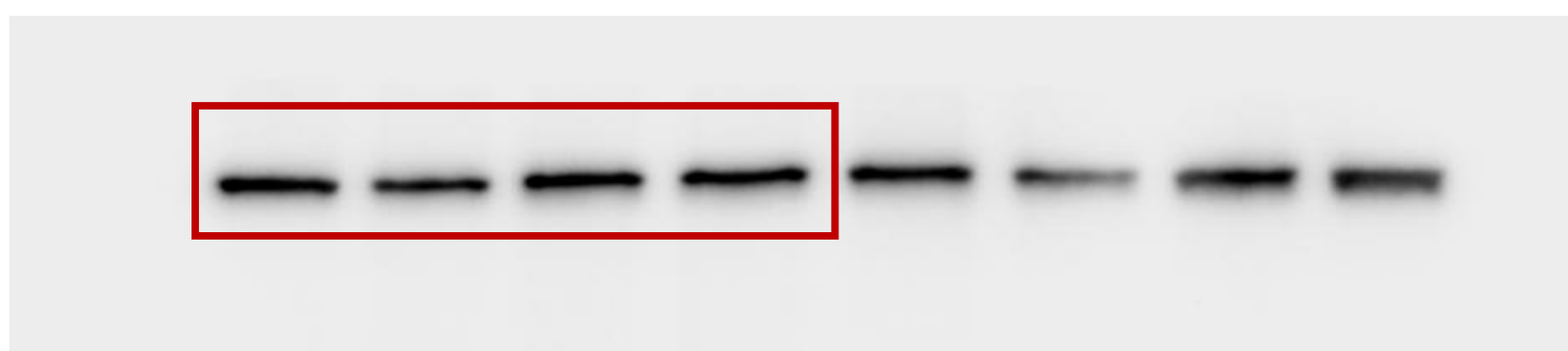

ACTIN

4C

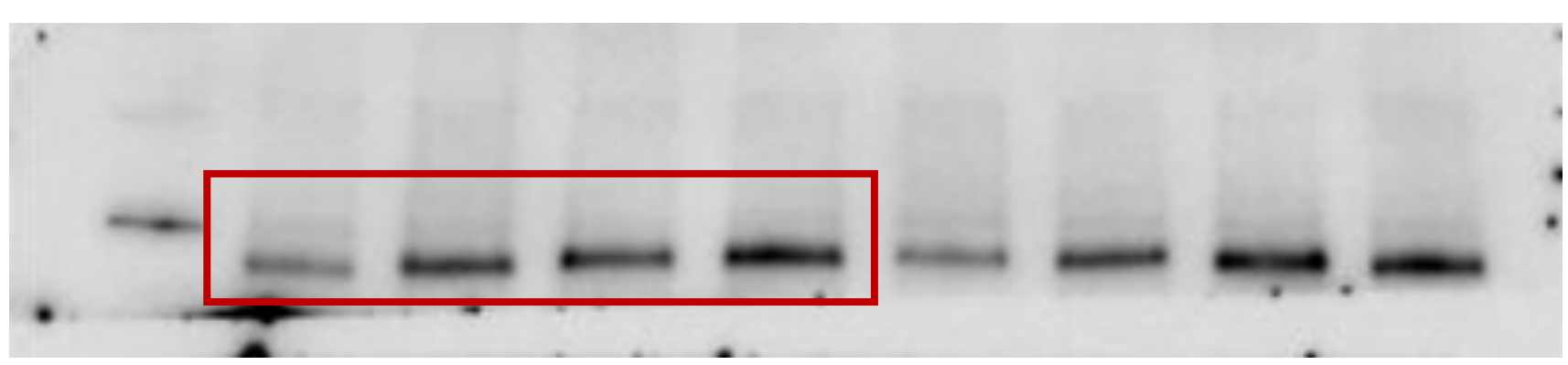

SMURF1

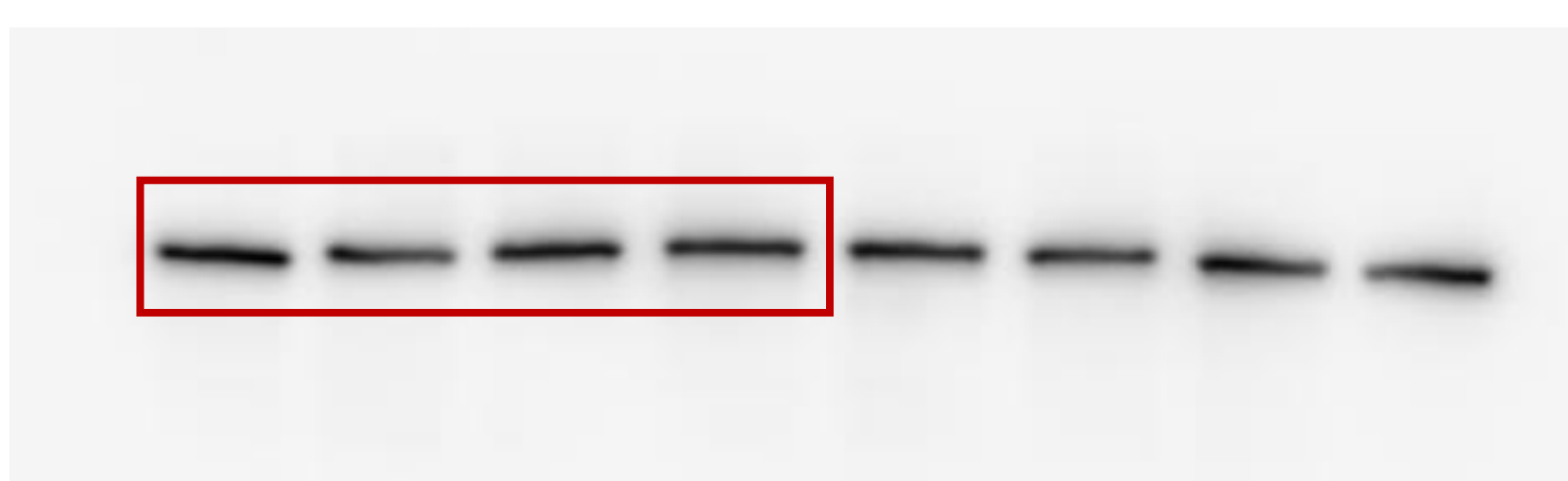

ACTIN

Full unedited gels for Figure 6B

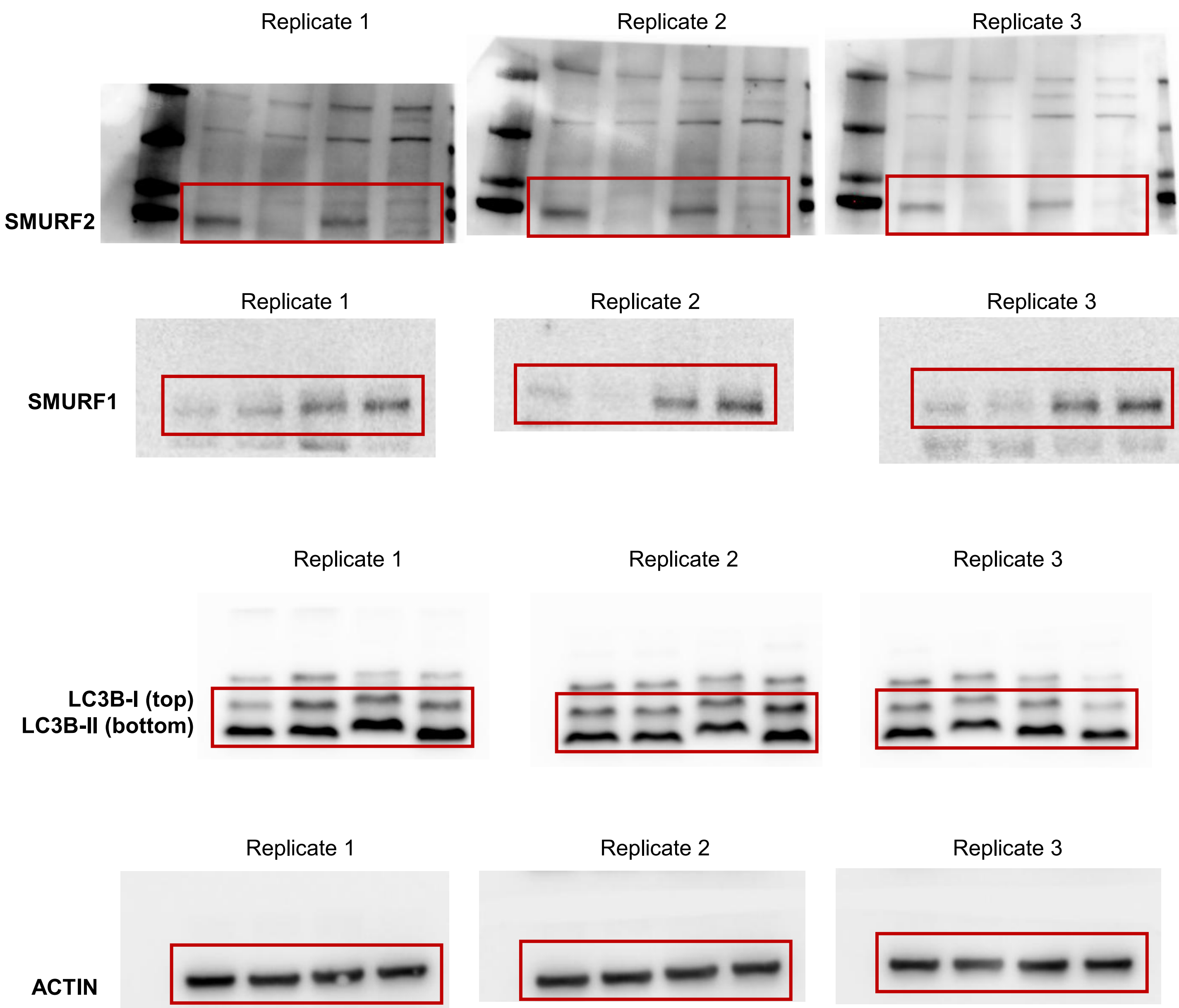

Full unedited gels for Figure 8F

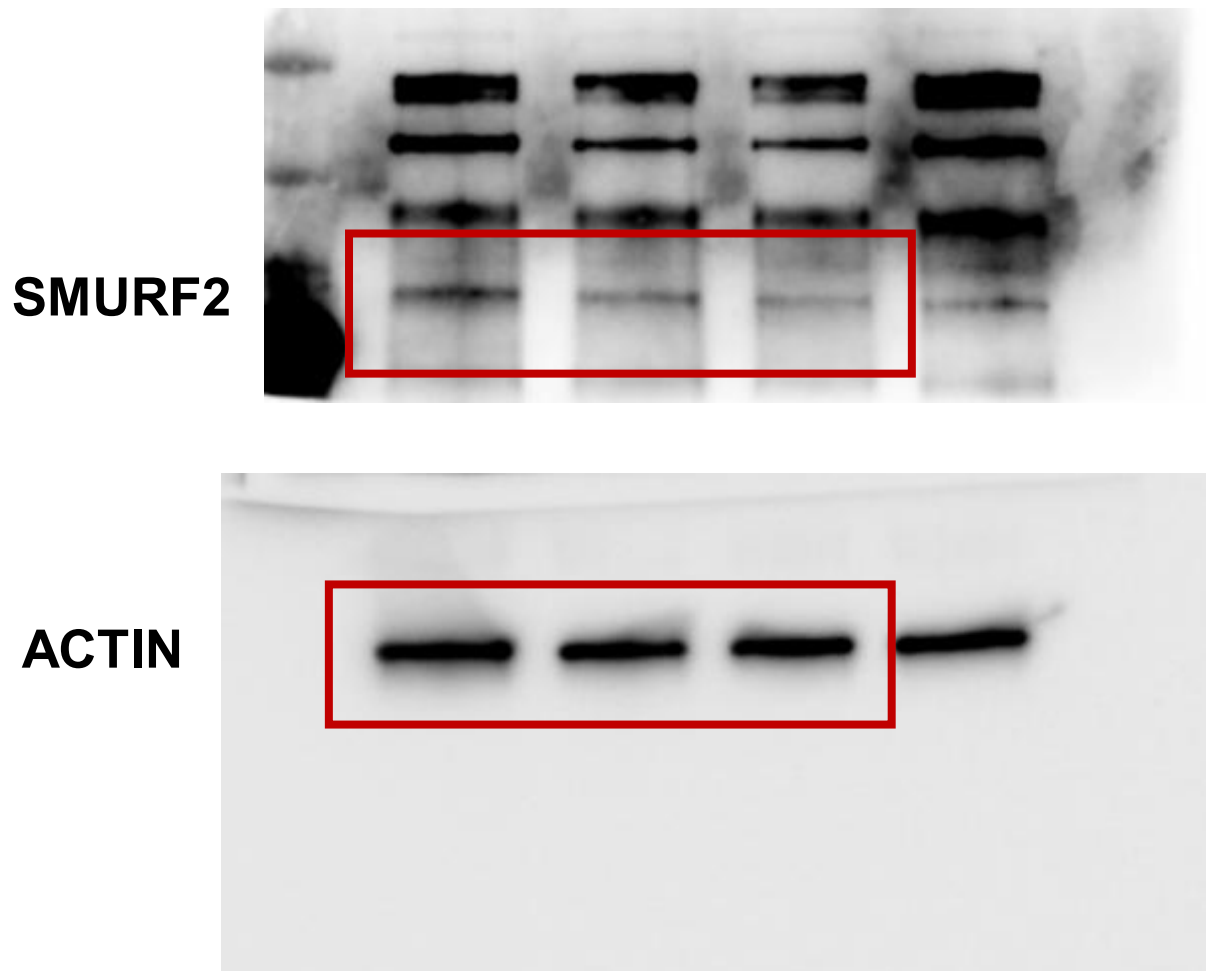

Full unedited gels for Figure S2A

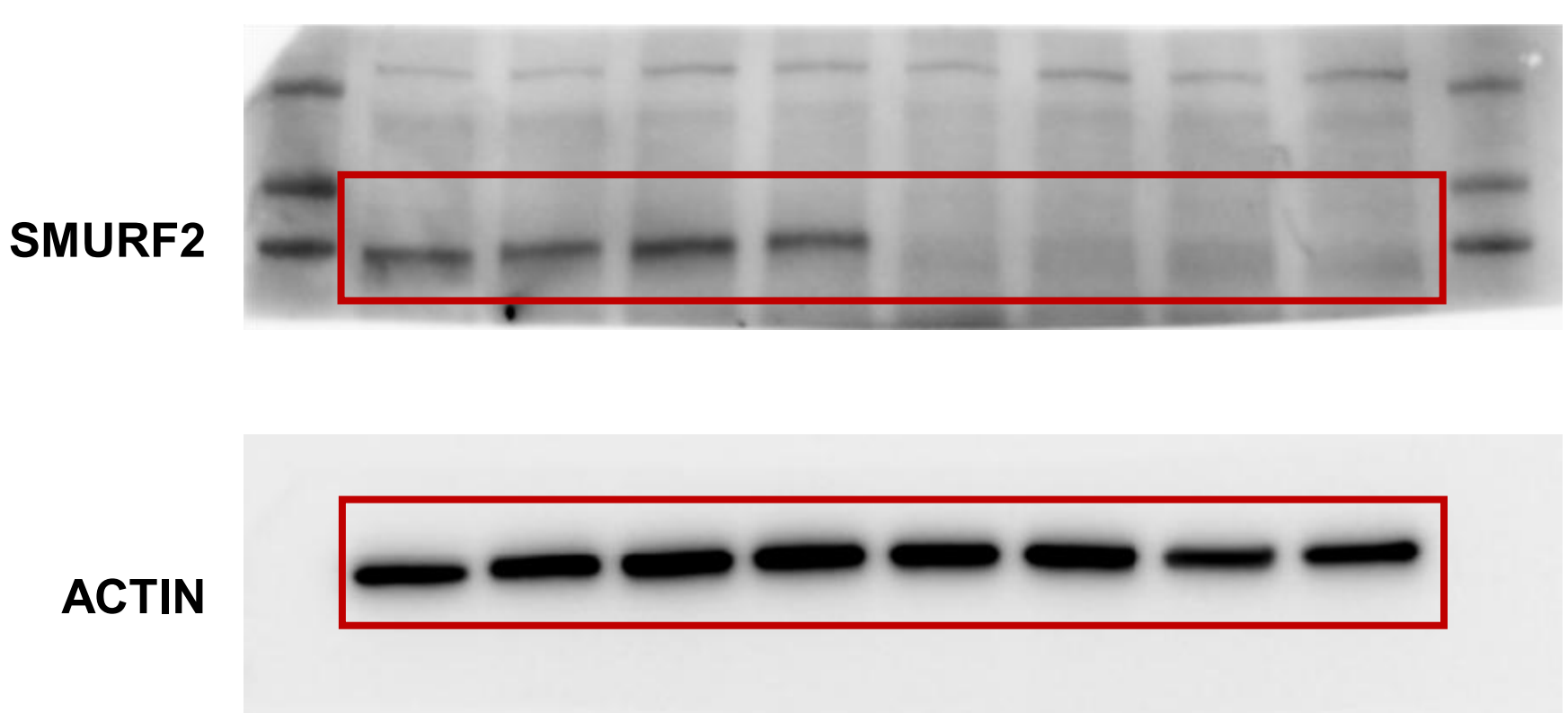

Full unedited gels for Figure S3C

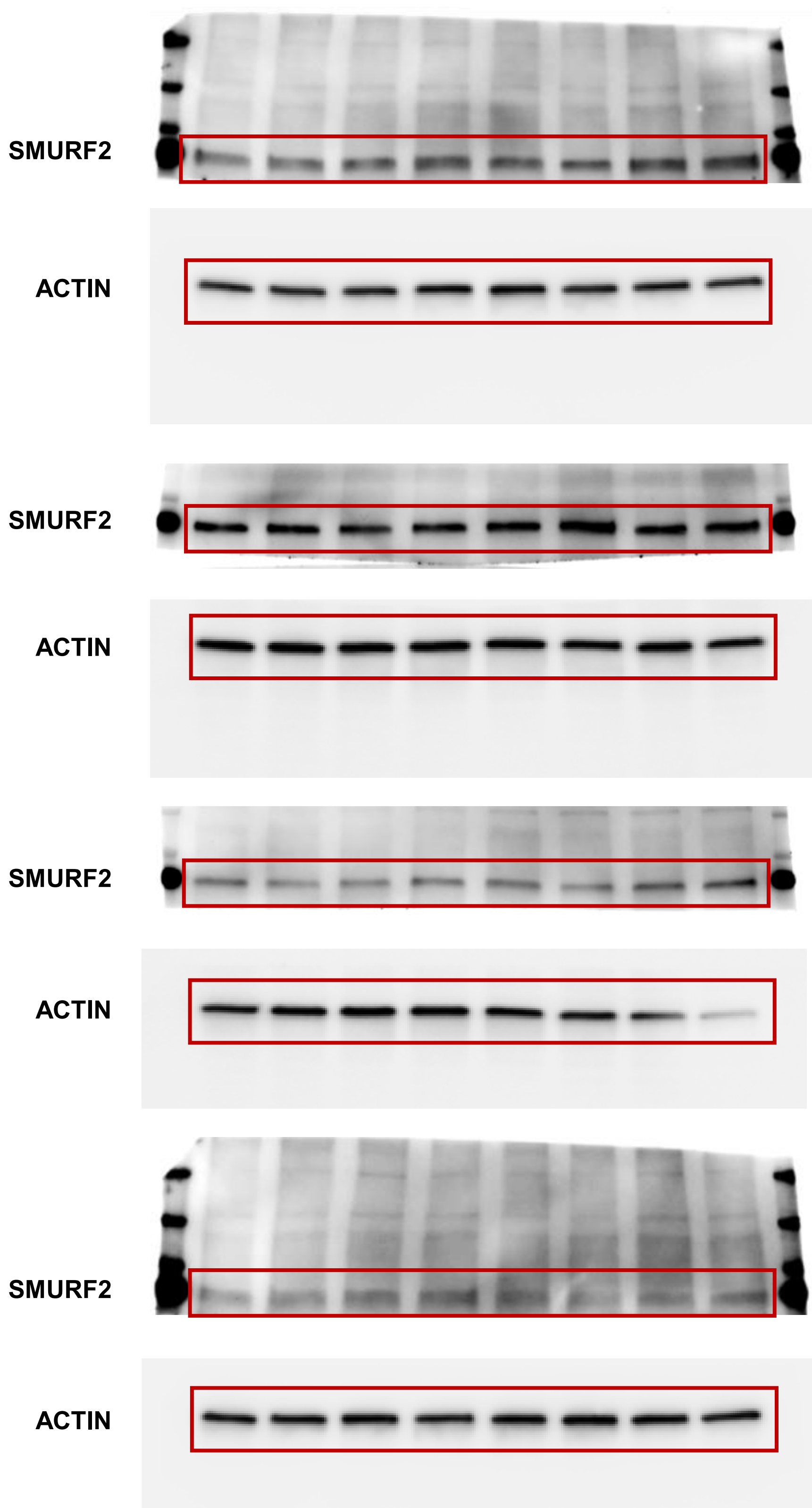

Supplement: Supplementary file 2 [file jciinsight-11-199853-s127.pdf]
